# Ferroelastic Control of the Multicolor Emission from a Triply Doped Organic Crystal

**DOI:** 10.1021/jacs.4c03190

**Published:** 2024-06-11

**Authors:** Patrick Commins, Marieh B. Al-Handawi, Caner Deger, Srujana Polavaram, Ilhan Yavuz, Rachid Rezgui, Liang Li, K. N. Houk, Panče Naumov

**Affiliations:** † Smart Materials Lab, New York University Abu Dhabi, PO Box 129188, Abu Dhabi, UAE; ‡ Department of Physics, Marmara University, Istanbul 34722, Türkiye; § Department of Chemistry and Biochemistry, University of California, Los Angeles, Los Angeles, California 90095-1569, United States; ∥ Department of Sciences and Engineering, Sorbonne University Abu Dhabi, PO Box 38044, Abu Dhabi, UAE; ⊥ Center for Smart Engineering Materials, New York University Abu Dhabi, PO Box 129188, Abu Dhabi, UAE; # Research Center for Environment and Materials, Macedonian Academy of Sciences and Arts, Bul. Krste Misirkov 2, Skopje MK-1000, Macedonia; ∇ Molecular Design Institute, Department of Chemistry, New York University, New York, New York 10003, United States

## Abstract

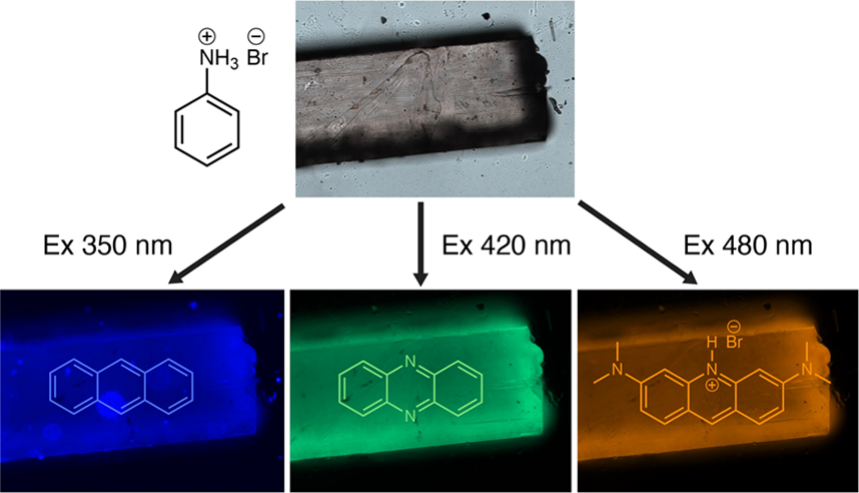

Emission from crystalline organic solids is often quenched by nonemissive energy-transfer deexcitation processes. While dispersion of fluorophores in polymers or other hosts has been used to enhance the emission intensity, this strategy results in randomization of guest orientation and optical losses at grain boundaries. Here, we report the doping of inherently nonemissive single crystals of anilinium bromide with three fluorescent organic molecules. The doping process equips the crystal with emission characteristics that tune from blue to deep orange. The emission intensity can be reversibly modulated by ferroelastic twinning, which causes the material to function as a multiemissive force sensor. This approach opens up new pathways in the manipulation of emissive properties in organic crystals and may have substantial implications for optoelectronic devices and sensors.

## Introduction

Luminescent materials represent a critical element in the quest for advanced materials. Common emissive organic devices such as light-emitting diodes are normally prepared as powders and incorporated into powder matrices, which are compatible with the common preparation procedures and mechanically comply with demanding sophisticated electrical designs.^1−3^ Organic crystals, having predefined and brittle shapes, would appear to be an unlikely scaffold for emissive materials. However, having ordered and tightly packed lattices, they are capable of anisotropic emission. They can host a range of impurities such as small molecules, where the matrix-isolation effect has been shown to enhance or modulate emission.^4−7^ Piezochromic and mechanochromic materials have also proven to be valuable force-sensitive emissive materials, although their emission is determined by their inherent chemical and crystal structure.^8−12^ The addition of foreign molecules into crystalline lattices can also serve as another, more general approach to intentionally modulate solid-state properties. Embedding exogenic molecules into biogenic or synthetic crystals has been established as a robust approach to eliciting new properties into crystalline materials.^13−19^ Once a guest species has been included into a host matrix, it remains physically and chemically inaccessible; however, its photophysical properties can still be externally modulated.

Here, we report ferroelastic twinning of an organic crystal that can reversibly tune the emission intensity of three fluorescent molecules embedded in the nonemissive single crystal by using external mechanical force. Specifically, the insertion of small amounts of anthracene (**A**), phenazine (**P**), and acridine orange (**O**) allows single crystals of anilinium bromide (**1**) to become emissive across a broad range of wavelengths in the blue, green, and orange regions of the spectrum. Upon application of force, the doped crystals (**APO@1**) undergo reversible ferroelastic twinning that causes the occluded dopants to reorient and thereby alter their emission. The unique mechanical responsiveness of these crystals opens up innovative opportunities in the design of force, pressure, or strain sensors similar to other dynamic crystals.^20−25^ The ability to adjust their luminescence in response to mechanical stress provides a new approach to sensing—one that offers a direct optical readout rather than relying on changes in electrical properties.^26−28^ The insights reported here offer a starting point for further investigation into mechanically controllable luminescence, providing a guiding principle for future explorations. We anticipate that this will pave the way for the development of novel optoelectronic and sensing technologies, underpinning the design of innovative materials and devices that can respond to and adapt to their environment.

## Results and Discussion

### Emissive Properties and Color of the Doped Crystals

**Figure 1:**
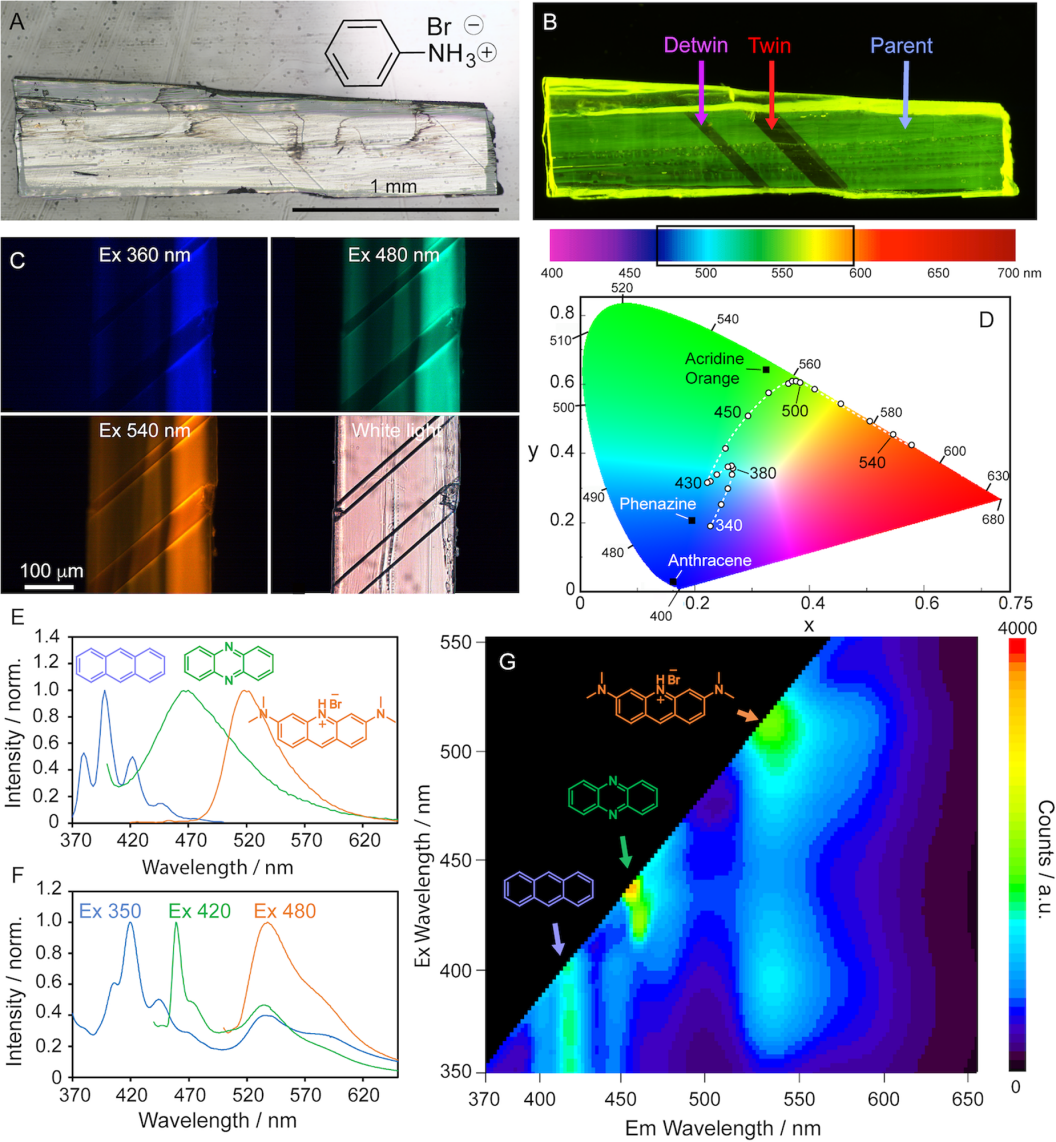
Broadband emission and spectra of **APO@1** crystals. (A) Optical image of an **APO@1** crystal in white light. Scale bar, 1 mm. (B) Polarized fluorescence image of an **APO@1** crystal irradiated with 350–380 nm light with its three domains: “parent” (before twinning), “twin” (after twinning), and “detwin” (after detwinning). (C) Color of the crystal when irradiated at 360, 480, and 540 nm and under white light (the collection windows were set to 430–470, 500–540, 560–580, and 400–700 nm, respectively). Scale bar, 100 μm. (D) Emission of the crystal excited in 10 nm intervals between 340 and 550 nm shown on the 1931 CIE color space. The emissions of anthracene (**A**), phenazine (**P**), and acridine orange (**O**) in methanol are added to the CIE plot for comparison, and the excitation wavelength is labeled next to the data point. A dotted white line is added to guide the eye. A visible spectrum chart is shown above the CIE plot, with a black box to indicate the emission range of **APO@1**. (E,F) Normalized emission spectra of **A**, **P**, and **O** in methanol (E) and **APO@1** (F) excited at 350, 420, and 480 nm. (G) 2D excitation–emission spectrum of **APO@1** powder showing the emission contribution of each of the three guests at varying excitations.

Single crystals of the doped material, **APO@1**, were grown by dissolving aniline and anthracene with acridine orange in 48% hydrobromic acid and methanol, and slow evaporation of the solution over 48 h. The phenazine was spontaneously formed by oxidation of the aniline *in situ* (for details, see the Methods section in the SI). We hypothesized that mildly substituted derivatives of phenazine could be effectively incorporated into the crystal structure. This reasoning led us to select anthracene and acridine orange. Beyond their structural resemblance to phenazine, the emissive characteristics of these two fluorophores cover a large region of the visible spectrum and allowed us to tailor the emissive properties of the **APO@1** crystals. The prismatic crystals were 0.5–50 mm long and translucent, with a faint pink discoloration (Figure 1A). X-ray diffraction structure analysis confirmed that the crystals were of the monoclinic *P*2_1_/*m* polymorph out of the several known polymorphs of **1**.^29,30^ The crystal structure of anilinium bromide is historically interesting and additional information on its structure was published previously.^29−31^ We confirmed by powder X-ray diffraction that unground crystals of **APO@1** are from the same polymorph (Figure S1). However, the crystals undergo a grinding-induced phase transition from the *P*2_1_/*m* phase to the orthorhombic *Pmmn* phase. Photoexcitation of the crystals with light across the ultraviolet (UV) and the visible spectral ranges confirmed the presence of the dopants in the bulk of the otherwise nonemissive crystal of **1**, which was surprisingly accommodating and incorporated all three emissive guests (**A**, **P**, and **O**; Figure 1). The presence of the emissive molecules is uniform within the bulk of the crystal and they are not simply deposited on the surface. Using confocal fluorescence microscopy, a *z*-scan image collected at varying depths of a crystal showed uniform fluorescence in the *x*, *y*, and *z* directions (Figure S2). The approximate concentration of the guests was determined by dissolving the **APO@1** crystals and measuring the fluorescence emission intensity of each guest. The guest concentrations were found to be 496 ± 16 nM, 56.9 ± 34.2 μM, and 281 ± 62 nM or 0.0025 ± 0.0001, 0.272 ± 0.163, 0.0021 ± 0.0004% of the mass of the **APO@1** crystals (m/m) for **A**, **P**, and **O**, respectively (Figure S3).

Excitation of the **APO@1** crystals at 360, 480, and 540 nm resulted in emission of blue, green, and orange light, respectively (Figure 1C, Movie S1). The presence of the dopants was further confirmed by fluorescence spectroscopy and mass spectrometry (Figure 1E,F, and Figure S4). The emission of the individual guests in **APO@1** at 350, 420, and 480 nm matches reasonably well the spectra of the individual emitters in methanol (Figure 1E,F). The absorption and emission spectra and molar absorptivity of **A**, **P**, and **O** are shown in Figure S5. The red-shift of the three most prominent emission peaks of **A** at 407, 420, and 444 nm in the **APO@1** crystals from the respective values in methanol at 380, 398, and 422 nm is attributed to dipole-ion interactions.^32^ The emission of **P** with a maximum at 460 nm and a shoulder at 476 nm in **APO@1** is similar to its solution spectra, having a maximum at 470 nm, though the reduced emission of the 476 nm peak in **APO@1** suggests energy transfer between **P** and **O**. Comparing the absorption spectra of acridine orange to the emission of **APO@1** shows good overlap between the donor and the acceptor, and further supports energy transfer between the two (Figure S6). The energy transfer between **P** and **O** was confirmed by comparing the lifetime of **P** in **APO@1** (1.741 ± 0.004 nm) to the lifetime of **P** in **P@1** (1.935 ± 0.006 ns) (Tables S1 and S2). The reduced lifetime of **P** in the presence of **O** indicates an energy transfer of 9.8%. The energy transfer was also observed by comparing the emission spectra of **APO@1** to those of **P@1** (Figure S7). The emission of **P@1** has a maximum at 476 nm and two shoulders at 461 and 506 nm, while in **APO@1**, the maximum is at 460 nm and there is a shoulder at 471 nm. The reduced intensity of the higher wavelength emission at 471 and 506 nm in **APO@1** confirms the energy transfer of **P** to **O**. Additionally, phenazine undergoes aggregation-induced quenching in solution (Figure S3C), though it readily emits from **APO@1** crystals (Figure 1F,G), showcasing the utility of isolating emitters into matrices to minimize the aggregation effects. Even though **P** was formed *in situ* in the presence of hydrobromic acid during crystallization, its spectrum peaks at 470 nm in **APO@1** and indicates emission from its neutral form rather than the protonated one, which has a peak centered at 517 nm (Figure S8). The guest **O**, excited at 480 nm in **APO@1**, has a maximum at 537 nm and a shoulder at 588 nm, and these spectral features are consistent with its solution spectrum, with a maximum at 522 nm. The enhancement of emission shoulders in **P** and **O** is likely a result of steric confinement in the solid state, which selectively strengthens transitions from certain excited states.^33^ A direct comparison of the emission of **APO@1** can also be made by observing the emission of **A@1**, **P@1**, and **O@1** in Figures S7 and S9.

The 2D excitation–emission plot in Figure 1G shows that the light emitted by the **APO@1** crystals depends strongly on the excitation wavelength since its emission can stem from one or more of the guests. For example, at excitations >480 nm, only **O** in **APO@1** absorbs and is the sole emitter; however, if the crystals are excited at 400 nm, all three guests contribute to the green color, as shown by the emissions at 420 nm (**A**), 468 nm (**P**), and 538 nm (**O**) (Figure 1G). This enables tuning of the emission of the crystal across the global color space, as illustrated in the 1931 CIE plot and the rainbow color strip in Figure 1D, where the color coordinates of **APO@1** crystals are shown for the crystals irradiated in 10 nm increments between 340 and 550 nm (the emission of three guests individually in methanol are also shown for comparison). When excited at 340 nm, the crystal emits blue light from **A**, which becomes greener as the excitation approaches 380 nm as there is an increasing contribution from **O** (Figure 1G). With excitation at 430 nm, the emission becomes blue/teal and comes from **P** and **O**. Above 480 nm, the emission has a greenish color, which gradually changes to deep orange at 550 nm from that of **O**.

### Effect of the Ferroelastic Twin Deformation on the Fluorescence Emission

**Figure 2:**
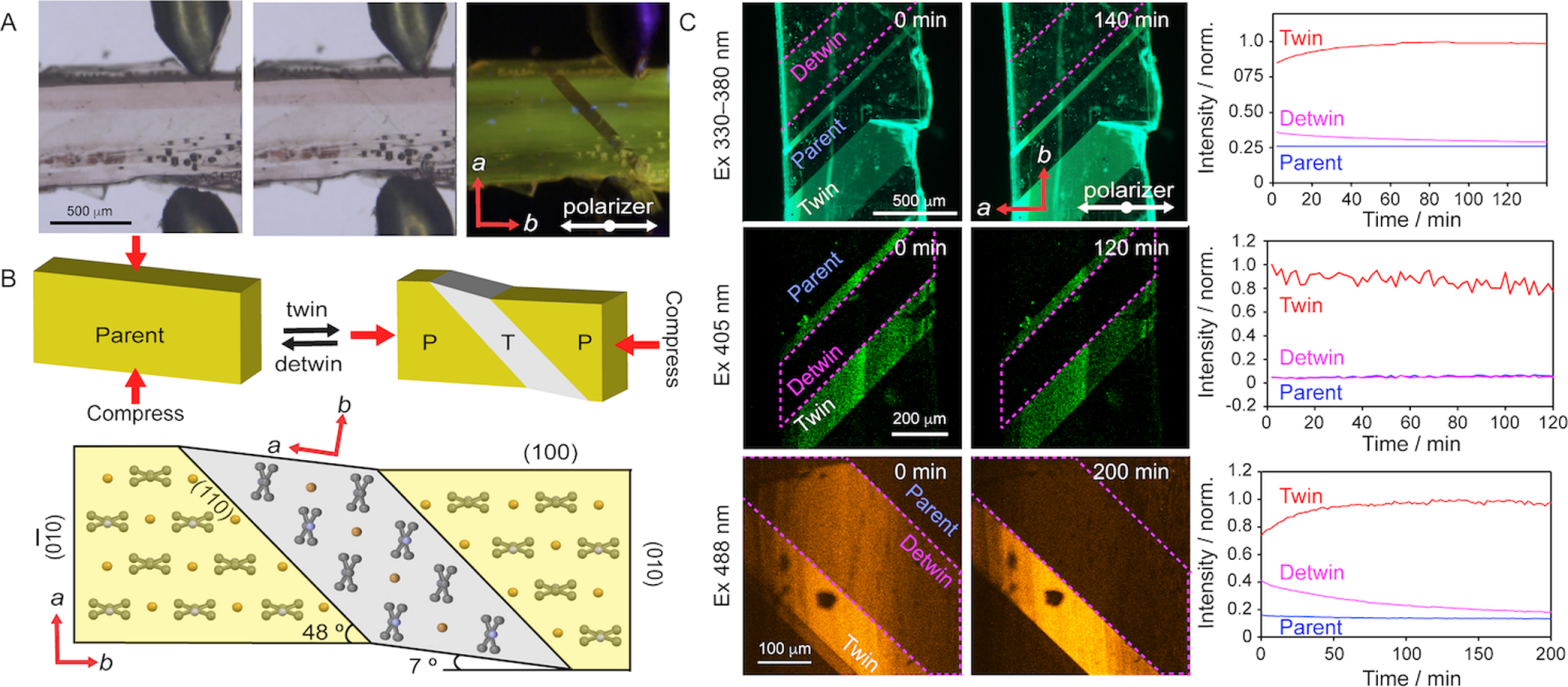
Twinning and detwinning, and their effect on the fluorescence of **APO@1** crystals. (A) Pair of micromanipulators twinning an **APO@1** crystal under polarized white light and UV light. (B) Change of molecular orientation in the structure of the host crystal **1** upon twinning and detwinning, as viewed along the *c* axis. Here, ‘P’ stands for a parent domain, and ‘T’ for a twinned domain. (C) Fluorescence recovery over time of each domain (parent, twinned, detwinned) monitored under excitation with 330–380 nm (top), 405 nm (middle), and 488 nm (bottom) light. The respective plots of time-dependent intensity are shown on the right. Note that the fluorescence recovered with excitation at 330–380 and 480 nm, but not at 405 nm.

If the **APO@1** crystals are compressed on their (100)/(1̅00) face, similar to the crystals of pure **1**,^34^ they can undergo a ferroelastic twin deformation that appears as a visible boundary running at 48° in respect to the long side of the crystal (Figure 2A, Figure S10, and Movie S2). The ferroelastic twinning was also confirmed by mechanical testing and shows the diagnostic hysteresis in stress required to twin and detwin the crystal (Figure S11). The twinned domain is related to the parent domain by a 90° rotation about the crystallographic *c* axis, and its structural identity was confirmed by single crystal X-ray diffraction analysis (Figure 2B, Figure S12). It has been established that dyes embedded into crystals commonly align themselves anisotropically along the crystallographic axes^13^ and all three guest molecules in **APO@1** adopt preferred orientations aligned along the *b* axis, as shown by the emission intensity in the parent and twin domains in Figure 2A. As also shown in Figure 2A, the guest molecules in the parent domain are aligned with the polarizer and thus the emission is bright yellow. In contrast, the guest molecules in the twinned region rotate together with the space-restrictive lattice of the host; they are crossed to the microscope polarizer, and that domain has a reduced emission. The twinned domain can be reverted to its original orientation (detwinned) by applying force onto the (010)/(01̅0) face (Figure 1B, Movie S2). The detwinned region is the same phase as the parent, and the two are indistinguishable by single crystal X-ray diffraction. Its worth noting that precise control over the area of the twin domain can be easily achieved by controlling the force applied on the crystal. When a single crystal was affixed to a tensile tester and tension was applied at a specific rate, a precise control on the growth of the twin domain was attained (Figure S13).

We noticed that the realignment of the guest molecules by twinning/detwinning is reversible, but the emission of the detwinned domain gradually recovers over time (Figure 2C). The emission intensity is directly related to the orientation of the emitters, and when transition dipole moments of the emissive transitions are aligned with the light polarization plane, the emission is maximized. This enabled us to track the alignment of the emitters with respect to the polarizer by measuring their fluorescence intensity over time. In Figure 2C, the twinned domain appears as a bright strip running diagonally across the crystal for all excitation wavelengths and intensity that changes over time. For excitations at 330–380 and 488 nm, corresponding to excitation of **A** and **O**, respectively, the intensity in the twinned region gradually increases and reaches a plateau over 100 min, indicating that the guest molecules reorient and become mostly *parallel* with the *b* axis and the polarized light plane. However, with excitation of 405 nm, which excites mostly **P**, the intensity of the emission from the twinned domain remains nearly constant over time, indicating that the guests are stationary. Similar reorientation of the emissive guests was also observed for the detwinned regions, where the intensity for excitation at 300–380 and 488 nm slowly decreases as a result of realignment of the guests with the *b* axis of the parent crystal, which is *perpendicular* to the polarized plane direction in the parent/detwinned domain. The change in fluorescence intensity in both twinned and detwinned regions reveals that not only are the guest molecules preferentially oriented within the host lattice but they also are unexpectedly dynamic.

### Locating the Guests with Polar Plots and Computational Analysis

**Figure 3:**
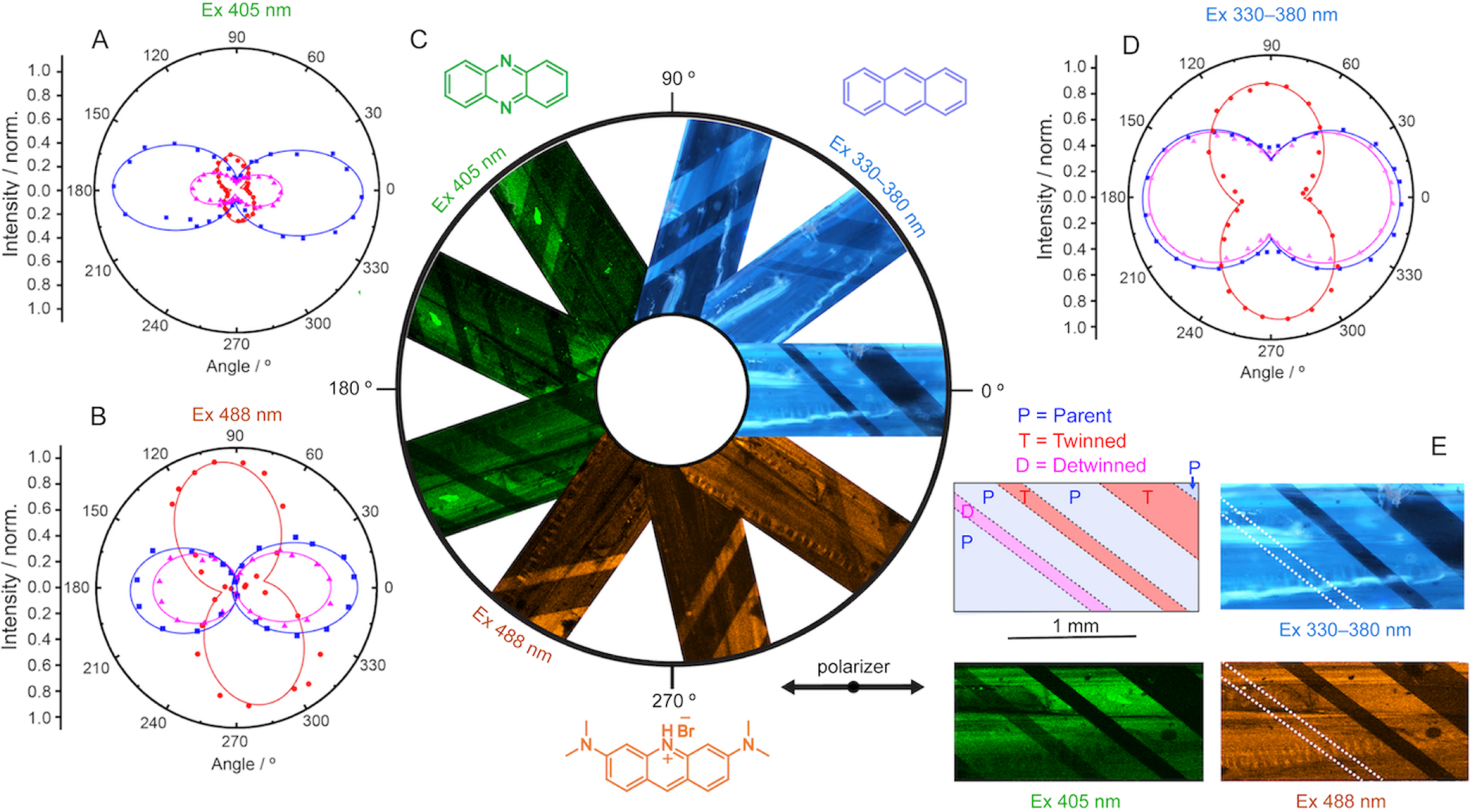
Polar plots of an **APO@1** crystal before and after twinning. (A, B, D) Polar plots of the crystal excited at 405, 488, and 330–380 nm, respectively. The blue, red, and magenta lines correspond to the parent, twinned, and detwinned domains. The elliptical lines have been added to guide the eye. (C) Polarized optical and confocal images of the crystal excited at 405, 488, and 330–380 nm and rotated at various angles to show the changing emission of each domain at different angles. The polarization of the incident light source is parallel to 0/180°. (E) Illustration of the crystal showing the location of the domains. The detwinned domain is highlighted with a dotted white line.

The very low concentration of the emissive guests in **APO@1** of less than 0.3 wt % (Figure S3) precludes direct determination of their structure by crystallographic means. Instead, the anisotropy of their emission was used to identify their orientation in the parent, twinned, and detwinned domains in the same crystal by using polarized confocal fluorescence microscopy. The excitation at 330–380, 405, and 488 nm was selected to excite predominantly one of the guests (Figure 2C, movie S3). The preferred orientation of the guest molecules appears as directional polar plots whose maxima align with the orientation of the transition dipole moments of the relevant electronic transitions^35,36^ (Figure 3, Figure S14). A series of images of an **APO@1** crystal rotated in 360° are shown in Figure 3C, and the parent, twin, and detwinned domains are labeled in blue, red, and magenta, respectively (Figure 3E).

The three polar plots at different excitation wavelengths (Figure 3A,B,D) show that all three guests have maximum emissions at *ca*. 178 and 358° for the parent and detwinned domains, and *ca*. 98 and 278° for the twinned domains, confirming that all guests have their transition state dipole moments approximately parallel to the *b* axis. Considering that the guests have flat, aromatic, anthracene-like structures, it is not surprising that they occupy nearly the same positions in the crystal lattice of **1**. After twinning, however, the emission of the guests varies at different excitation wavelengths. The plots at excitations of 330–380 nm coming mostly from **A** and 488 nm from **O** (Figure 3B,D) have a maximum emission intensity that is similar for the parent, twinned, and detwinned regions; there is only a reduction of 6% (330–380 nm) and 27% (488 nm) from the maximum intensity. The emission intensity at 405 nm (Figure 3A), which is mostly due to the emission of **P**, however, is significantly decreased by 58% in the twinned domain and by 65% in the detwinned domain. The reduced emission intensity signifies that the molecules of **P** become more disordered after each perturbation of the host lattice.

**Figure 4:**
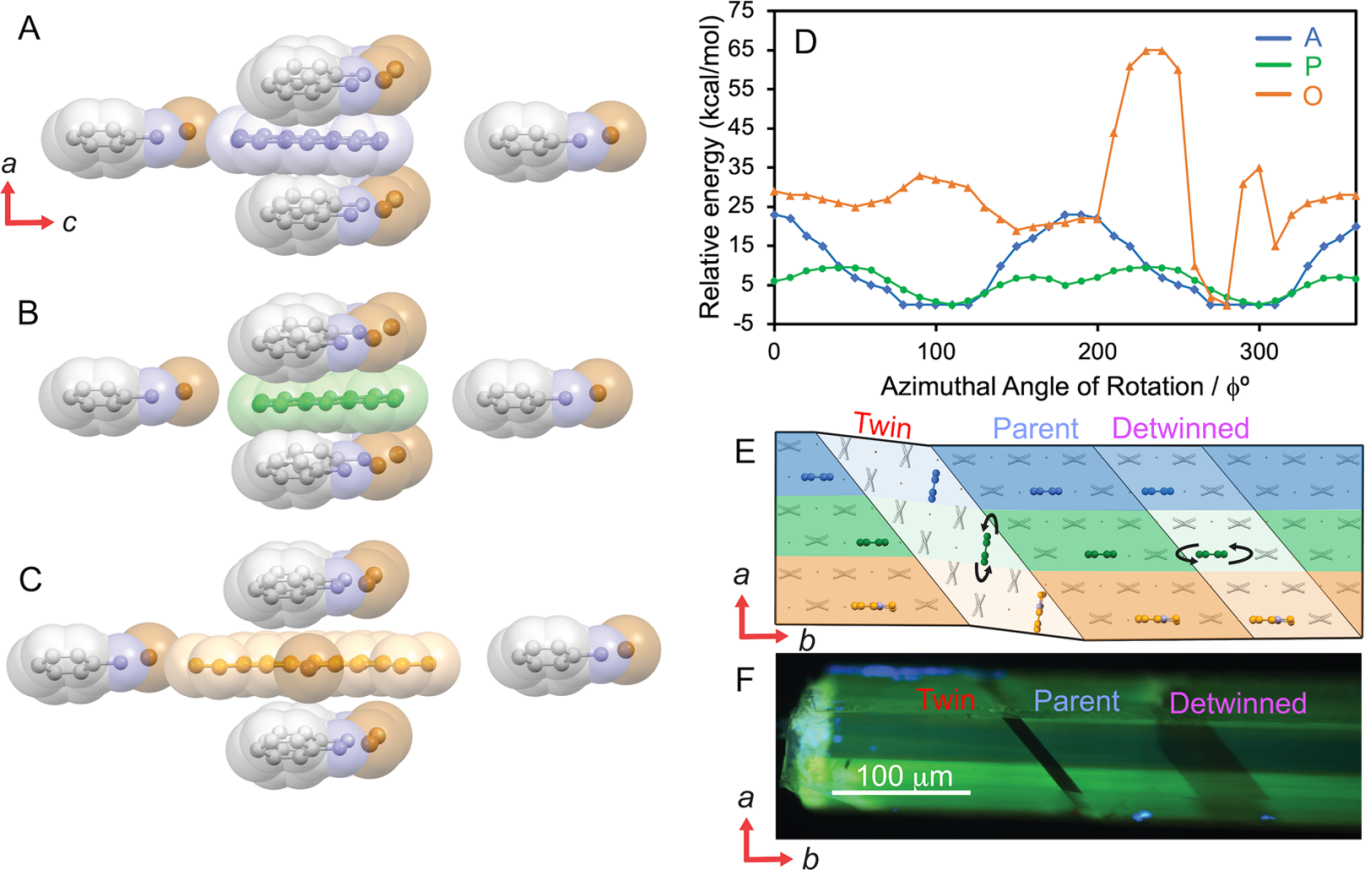
Orientation of the guest molecules in the host lattice. (A, B, C). Models of guest **A** (A), **P** (B), and **O** (C) inserted into the lattice of anilinium bromide crystal structure and surrounded by 6 adjacent anilinium bromide ion pairs. The **A**, **P**, and **O** molecules are placed at an azimuthal rotation of 0° in these models. The guest was azimuthally rotated about the *a* axis, and the relative energy was calculated by using the B3LYP/6-311G(d,p) method. (D) Relative energy diagram plot for the three guests at varying azimuthal rotations showing that **A** and **P** have minima near 100° while **O** has a minimum neat 270°. The blue, green, and orange lines correspond to **A**, **P**, and **O**, respectively. (E) Representation of the *ab* plane showing the calculated location **A** (blue), **P** (green), and **O** (orange) in the parent, twinned, and detwinned domains. There are black arrows near the **P** molecules in the twin and detwinned domains to indicate it is disordered. (F) Polarized optical image of **APO@1** crystals on the *ab* plane is shown for comparison.

To better locate the orientation of the guests within the crystal, which should account for the different trends in emissions of different guests over time, we performed first-principles calculations based on density functional theory (DFT) at the B3LYP/6-311G(d,p) level.^37^ The guest molecules were placed into the host lattice by first removing two molecules of anilinium bromide and then laying the guest flat along the *b* axis into the vacancy followed by allowing the assembly to structurally relax. The lowest energy structure was determined based on their azimuthal rotation along the *a* axis. The resulting relative energy diagram for each guest at varying azimuthal rotations of the guest is shown in Figure 4A–C. The relative energy profile for **A** displays a relatively flat energy potential from 80–120° (Figure 4D), indicating that the structural accommodation of the molecule in the lattice is associated with low energy. **P** is slightly stabilized at a 110° orientation, though a rotation of 100 or 120° would only be 0.54 and 1.01 kcal/mol higher in energy, respectively. The bulkiest molecule, **O**, has a strong preference for a 260–270° azimuthal rotation, and a slight rotation in either direction causes the model to increase more than 35 kcal/mol in energy. The three energy profiles show that each of the emitters has a preferred orientation that places its transition state dipole moment along the *b* axis of the anilinium bromide crystal and is in good agreement with the polar plots shown in Figure 3A,B,D. The energy profiles also hint at why the emission intensity decreases with excitation at 405 nm in Figure 3A. Molecule **P**, which is a significant contributor to the emission by excitation at this wavelength, has a relatively flat energy profile with only a 9 kcal/mol energy difference between its highest and lowest orientations. Hence, the energy cost of having the guest molecules at a nonideal orientation is relatively low. Additionally, there are two local minima at 0° and 180° that **P** may also inhabit. The opposite is true for **O**, and the energetic cost of lying at its non-optimal geometry is ca. 35 kcal/mol. This relatively deep energetic well may explain why the fluorescence of **O** can recover in Figure 2C, as there is a strong thermodynamic reason for the molecules to return to their preferred orientation.

Corroborating the information from the computational analysis and polar plots has allowed us to construct a model identifying the location of each guest in each of the domains in the **APO@1** crystal (Figures 4E) where the blue, green, and orange regions correspond to the **A**, **P**, and **O** molecules, respectively. In the parent domain, all three guests lie with their transition state dipole moments primarily along the *b* axis. When the crystal is twinned, most of the molecules rotate approximately 83° with the anilinium bromide lattice; however, some of the molecules of **P** become disordered, as evidenced by the decreased emission intensity in the polar plots (Figure 3A). When the crystal is detwinned, a majority of **A** and **O** molecules return to their original orientation, though some become disordered and eventually realign over time with the *b* axis, as shown by the recovered fluorescence intensity in Figure 3B,D. However, the molecules of **P** become increasingly more disordered with each twining-detwinning cycle (Figure 4E).

### Application as a Photomechanical Force Sensor

**Figure 5:**
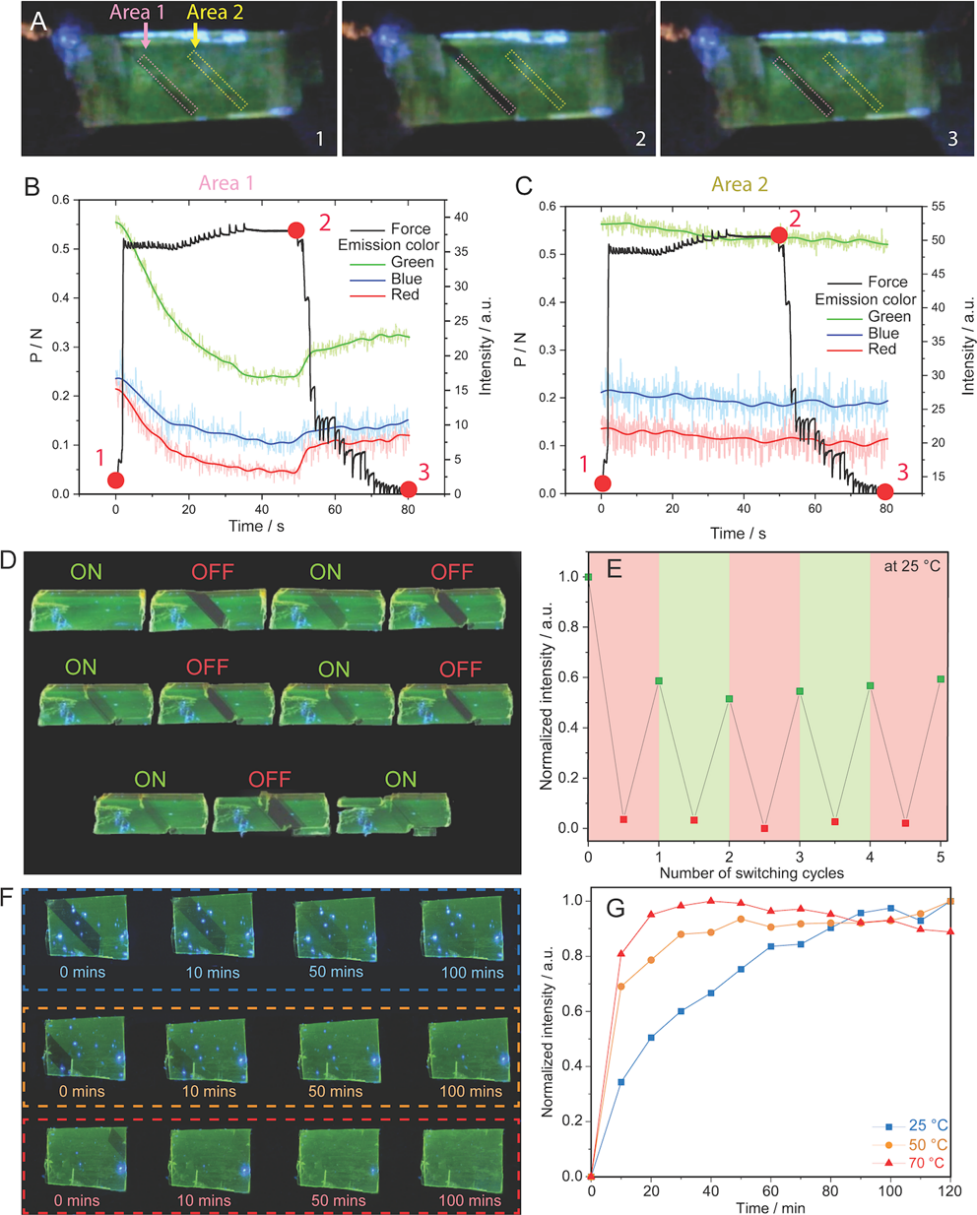
Photomechanical force sensing by using the **APO@1** crystals. (A) Polarized optical images showing a crystal of **APO@1** irradiated at 350–380 nm that was ferroelastically twinned and detwinned. The color of the emission was measured in area 1, a twinned region, and area 2, a region of the parent crystal. At *t* = 0 s (1), the crystal is at rest. After 3 s, the twin domain was formed and propagates. At *t* = 50 s the tension was terminated and the crystal was compressed until *t* = 80 s, whereupon it was completely detwinned. (B,C) Force-emission plots displaying the color of light emitted from area 1 and 2, as the crystal undergoes twin formation. The emission of red, green, and blue light all gradually decrease in area 1 (B) while the twin is being formed and slightly increase when the crystal is detwinned at 50 s. The emission color in area 2 (C) remains constant. The raw data for each color’s emission is shown as a shaded color, and the fitted data is represented by a bold solid colored line. (D) Optical polarized images of a crystal that was twinned and detwinned into its on/off states five times visualized under broadband UV light. (E) Maximum normalized intensity of the twin domain after each twinning/detwinning cycle. (F) Optical polarized images of a crystal that was detwinned and the fluorescence recovery was measured at 25, 50, and 70 °C. The crystal was irradiated with broadband UV light. (G) Rate of fluorescence recovery in the detwinned region measured at 25, 50, and 70 °C showing that the fluorescence recovers faster at higher temperatures.

The field of solid-state photoswitching devices is innately attractive as they have the ability to form high contrast readouts nondestructively. The field has been incorporating common organic photoswitches into various media such as liquid crystal polymers^38^ and hydrogels^39^ to achieve functional materials.^40^ However, the conformational freedom gained by introducing chromophores into an isotropic medium, such as a polymer, comes at a loss of control of its orientation. By incorporating three emitters into the **APO@1** crystal and using their ability to rotate within the twin domains, the crystals can serve as an all-organic real-time force sensors that change their emissions in response to force, similar to some inorganic materials.^41,42^ The force sensing ability of the **APO@1** crystals was investigated by constructing a force-emissivity plot (Figure 5A–C, Movie S4). A crystal of **APO@1** was adhered to a tensile tester and irradiated at 350–380 nm light, and the color of its emission was measured using a polarized microscope while the crystal was ferroelastically twinned and detwinned under tension and compression. At time zero (time point 1), the emissions of red, green, and blue light in both area 1 and area 2 in Figure 5A are at their maximum. Once a force of 0.51 N of tension was applied, a twin domain (area 1) was formed and it began propagating along the crystal for 50 s, which was accompanied by a drop in the red, green, and blue light by 80, 55, and 62%, respectively. After 50 s (time point 2), the tension was halted and the crystal was compressed to its original shape. During the compression, there was a slight jump in the emission at ca. 53 s as the lattice was detwinned and the red, green, and blue emission recovered to 50, 56, and 59% of their maximum over the next 30 s. The emission in area 2, the parent domain, remained relatively constant throughout. To the best of our knowledge, this is the first example of an organic multiemissive force sensor. A notable previous study has reported a superelastochromic crystal^43^ that shows emission change from yellow-green to yellow-orange in response to force. The change in the emission color is less sensitive for detection relative to the decrease in overall emission of the three guest molecules in **APO@1**. The sensor also has varying sensitivity as the inclusion of the three different emitters allows the material to have different sensitivities based upon which the wavelength of light is measured. **APO@1** is particularly sensitive in the red as there is an 80% decrease in its intensity from its baseline emission; however, it is less sensitive in the green as its intensity is only reduced by 55%. The data shows that **APO@1** crystals can detect forces as low as 0.51 N, which is intriguing as an organic light emitting force sensor but is less sensitive than some flexible sensors, which have shown to have a lower detection limit of >0.098 mN.^41,44^ The threshold force required to induce a twin domain and thus change its fluorescence is dependent upon the crystal’s size, where smaller crystals have lower threshold forces and larger ones have larger threshold forces. This relationship is displayed in Figure S15 and shows that the threshold force required to induce a twin can also be controlled by the crystal size.

The cyclability of the mechanoswitching behavior of the crystal’s fluorescence was tested by measuring its intensity under a polarized microscope over five cycles on a single crystal (Figure 5D,E). Upon twinning, the emission in the twinned domain was significantly reduced by approximately 95% when excited at 330–380 nm, putting it into an “off-state”. However, when the twinned domain was mechanically detwinned, the fluorescence intensity recovered by ∼60% within 35 min in the dark and returned to its “on-state”. This recovery of the intensity (∼60%) was repeated over five cycles with no loss of emission and showcased the crystal’s robustness. The photoswitching behavior of these crystals can also be controlled thermally. The emission recovery of a twinned and detwinned region was measured at 25, 50, and 70 °C (Figure 5F,G). The temperature-dependent experiments were performed on the same crystal to reduce possible variance across multiple crystals, while different locations of the crystal were twinned and detwinned at each temperature. At 25, 50, and 70 °C, 90% recovery of the emission was observed in the detwinned domain after 120, 50, and 20 min, respectively (Figure 5G). The results show that the crystal accelerates its recovery as the temperature increases. This further indicates that the reorientation of the guests into their preferred orientations is a thermodynamically favorable process, and so the rate of emission recovery is naturally hastened at higher temperatures.

## Conclusions

In summary, we have incorporated three different compounds with emissions that cover a large span of the visible spectrum into single crystals of the nonemissive host, anilinium bromide. The crystals can emit colors ranging from deep blue to orange depending on the incident light. In response to mechanical pressure, the resulting doped, transparent crystals reversibly form ferroelastic twin domains, and the guests move in tandem with the twinned and detwinned domains, which affords precise and reversible control over the spatial emission intensity of the emitters. We were able to locate the position of the guests inside the crystal using both computational and experimental methods and found that the emitters lie flat along the *b* axis of the host lattice. The controllable rotation of the guests within the crystal allows them to be used as a reversible photoswitch and multiemissive force sensor, with reduction of the emission in the twinned domain by 95% and restoration to 65% by detwinning within 35 min. This work showcases the effective manipulation and control of the optical properties of the emitters by embedding them into a crystalline matrix and reordering their orientation by simply using mechanical force.

## Supporting Information Available

Legends of Movies S1–S4 (PDF)
Crystal of **APO@1** emitting light of different colors (orange, green, blue), depending on the wavelength of the excitation wavelength MP4)
Pair of micromanipulators twinning and detwinning a crystal under polarized white light and UV light (MP4)
Fluorescence recovery over time of each domain (parent, twinned, detwinned) monitored under excitation with 330–380, 405, and 488 nm light (MP4)
Force/emission experiment measuring the color of light emitted in areas 1 and 2 of the crystal as it undergoes twinning (MP4)
Experimental details, materials, and methods (PDF)

## References

[ref1] Nair G. B.; Swart H. C.; Dhoble S. J. A Review on the Advancements in Phosphor-Converted Light Emitting Diodes (Pc-LEDs): Phosphor Synthesis, Device Fabrication and Characterization. Prog. Mater. Sci. 2020, 109, 100622 10.1016/j.pmatsci.2019.100622.

[ref2] Mehare M. D.; Mehare C. M.; Swart H. C.; Dhoble S. J. Recent Development in Color Tunable Phosphors: A Review. Prog. Mater. Sci. 2023, 133, 101067 10.1016/j.pmatsci.2022.101067.

[ref3] McKittrick J.; Shea-Rohwer L. E. Review: Down Conversion Materials for Solid-State Lighting. J. Am. Ceram. Soc. 2014, 97 (5), 1327–1352. 10.1111/jace.12943.

[ref4] Cradock S.; Hinchcliffe A. J. Matrix Isolation: A Technique for the Study of Reactive Inorganic Species; (Cambridge University Press, 1975) pp 1–99.

[ref5] Perutz R. N. Photochemical Reactions Involving Matrix-isolated Atoms. Chem. Rev. 1985, 85, 77–96. 10.1021/cr00066a001.

[ref6] Rawashdeh-Omary M. A. Remarkable Alteration of Photophysical Properties of Cyclic Trinuclear Complexes of Monovalent Coinage Metals upon Interactions with Small Organic Molecules. Comments Inorg. Chem. 2012, 33 (3–4), 88–101. 10.1080/02603594.2012.747958.

[ref7] Batra K.; Sinha N.; Kumar B. Sunset Yellow Dye Doped Ammonium Dihydrogen Phosphate Single Crystals with Enhanced Optical, Mechanical and Piezoelectric Properties. J. Mater. Sci.: Mater. Electron 2019, 30 (16), 14902–14912. 10.1007/s10854-019-01861-5.

[ref8] Dai Y.; Liu H.; Geng T.; Duan R.; Li X.; Liu Y.; Liu W.; He B.-G.; Sui L.; Wang K.; Zou B.; Yang B.; Qi Y. Piezochromic Fluorescence of Anthracene Derivative Crystals with Different Stacking Patterns Designed around Excimers. J. Mater. Chem. C 2023, 11 (14), 4892–4898. 10.1039/D2TC05535J.

[ref9] Zhao W.; He Z.; Peng Q.; Lam J. W. Y.; Ma H.; Qiu Z.; Chen Y.; Zhao Z.; Shuai Z.; Dong Y.; Tang B. Z. Highly Sensitive Switching of Solid-State Luminescence by Controlling Intersystem Crossing. Nat. Commun. 2018, 9 (1), 3044 10.1038/s41467-018-05476-y. 30072690 PMC6072740

[ref10] Zhang R.; Wang Q.; Zheng X. Flexible Mechanochromic Photonic Crystals: Routes to Visual Sensors and Their Mechanical Properties. J. Mater. Chem. C 2018, 6 (13), 3182–3199. 10.1039/C8TC00202A.

[ref11] Wei Y.; Yang R.; Cui G.; Dai S.; Pan G.; Wang J.; Ren H.; Ma W.; Gu Z.; Zhang C.; Li G.; Liu Z.; Xu B.; Tian W. Low-Pressure Sensitive Piezochromic Fluorescence Switching of Tetraphenylethylene-Anthraquinone. Chem.—Eur. J. 2023, 29 (48), e202301070 10.1002/chem.202302240. 37166756

[ref12] Lv Z.; Man Z.; Xu Z.; Fu L.; Li S.; Zhang Y.; Fu H. High Contrast and Bright Emission Piezochromic Fluorescence in Organic Crystals via Pressure Modulated Exciton Coupling Effect. Adv. Opt. Mater. 2021, 9 (17), 2100598 10.1002/adom.202100598.

[ref13] Kahr B.; Gurney R. W. Dyeing Crystals. Chem. Rev. 2001, 101 (4), 893–952. 10.1021/cr980088n. 11709861

[ref14] Kahr B.; Shtukenberg A. G. Dyeing Crystals since 2000. CrystEngComm 2016, 18 (47), 8988–8998. 10.1039/C6CE02185A.

[ref15] Pinsk N.; Wagner A.; Cohen L.; Smalley C. J. H.; Hughes C. E.; Zhang G.; Pavan M. J.; Casati N.; Jantschke A.; Goobes G.; Harris K. D. M.; Palmer B. A. Biogenic Guanine Crystals Are Solid Solutions of Guanine and Other Purine Metabolites. J. Am. Chem. Soc. 2022, 144 (11), 5180–5189. 10.1021/jacs.2c00724. 35255213 PMC8949762

[ref16] Gueta R.; Natan A.; Addadi L.; Weiner S.; Refson K.; Kronik L. Local Atomic Order and Infrared Spectra of Biogenic Calcite. Angew. Chem., Int. Ed. 2007, 46 (1–2), 291–294. 10.1002/anie.200603327. 17124701

[ref17] Estroff L. A.; Cohen I. Micelles in a Crystal. Nat. Mater. 2011, 10 (11), 810–811. 10.1038/nmat3156. 22020000

[ref18] Weber E.; Pokroy B. Intracrystalline Inclusions within Single Crystalline Hosts: From Biomineralization to Bio-Inspired Crystal Growth. CrystEngComm 2015, 17 (31), 5873–5883. 10.1039/C5CE00389J.

[ref19] Liu F.; Hooks D. E.; Li N.; Rubinson J. F.; Wacker J. N.; Swift J. A. Molecular Crystal Mechanical Properties Altered via Dopant Inclusion. Chem. Mater. 2020, 32 (9), 3952–3959. 10.1021/acs.chemmater.0c00433.

[ref20] Koshima H. Mechanically Responsive Materials for Soft Robotics; Wiley-VCH, 2020; pp 1–175.

[ref21] Awad W. M.; Davies D. W.; Kitagawa D.; Mahmoud Halabi J.; Al-Handawi M. B.; Tahir I.; Tong F.; Campillo-Alvarado G.; Shtukenberg A. G.; Alkhidir T.; Hagiwara Y.; Almehairbi M.; Lan L.; Hasebe S.; Karothu D. P.; Mohamed S.; Koshima H.; Kobatake S.; Diao Y.; Chandrasekar R.; Zhang H.; Sun C. C.; Bardeen C.; Al-Kaysi R. O.; Kahr B.; Naumov P. Mechanical Properties and Peculiarities of Molecular Crystals. Chem. Soc. Rev. 2023, 52 (9), 3098–3169. 10.1039/D2CS00481J. 37070570

[ref22] Naumov P.; Chizhik S.; Panda M. K.; Nath N. K.; Boldyreva E. Mechanically Responsive Molecular Crystals. Chem. Rev. 2015, 115 (22), 12440–12490. 10.1021/acs.chemrev.5b00398. 26535606

[ref23] Colin-Molina A.; Karothu D. P.; Jellen M. J.; Toscano R. A.; Garcia-Garibay M. A.; Naumov P.; Rodríguez-Molina B. Thermosalient Amphidynamic Molecular Machines: Motion at the Molecular and Macroscopic Scales. Matter 2019, 1 (4), 1033–1046. 10.1016/j.matt.2019.06.018.

[ref24] Commins P.; Hara H.; Naumov P. Self-Healing Molecular Crystals. Angew. Chem., Int. Ed. 2016, 55 (42), 13028–13032. 10.1002/anie.201606003. 27634399

[ref25] Li Y.-X.; Liu Z.-K.; Cao J.; Tao J.; Yao Z.-S. Stress-Induced Inversion of Linear Dichroism by 4,4′-Bipyridine Rotation in a Superelastic Organic Single Crystal. Angew. Chem., Int. Ed. 2023, 62 (11), e202217977 10.1002/anie.202217977. 36647773

[ref26] Amjadi M.; Kyung K.-U.; Park I.; Sitti M. Stretchable, Skin-Mountable, and Wearable Strain Sensors and Their Potential Applications: A Review. Adv. Funct. Mater. 2016, 26 (11), 1678–1698. 10.1002/adfm.201504755.

[ref27] Park K. S.; Baek J.; Park Y.; Lee L.; Lee Y.-E. K.; Kang Y.; Sung M. M. Inkjet-Assisted Nanotransfer Printing for Large-Scale Integrated Nanopatterns of Various Single-Crystal Organic Materials. Adv. Mater. 2016, 28 (15), 2874–2880. 10.1002/adma.201505594. 26891239

[ref28] Zhou L.; Jung S.; Brandon E.; Jackson T. N. Flexible Substrate Micro-Crystalline Silicon and Gated Amorphous Silicon Strain Sensors. IEEE Trans. Electron Devices 2006, 53 (2), 380–385. 10.1109/TED.2005.861727.

[ref29] Taguchi I. An X-ray Study on the Phase Transition of Aniline Hydrobromide, C_6_H_5_NH_3_Br. Bull. Chem. Soc. Jpn. 1961, 34 (3), 392–395. 10.1246/bcsj.34.392.

[ref30] Fu D.-W.; Gao J.-X.; Huang P.-Z.; Ren R.-Y.; Shao T.; Han L.-J.; Liu J.; Gong J.-M. Observation of Transition from Ferroelasticity to Ferroelectricity by Solvent Selective Effect in Anilinium Bromide. Angew. Chem., Int. Ed. 2021, 60 (15), 8198–8202. 10.1002/anie.202015219. 33480082

[ref31] Sakai T.; Terauchi H. Structure of Anilinium Bromide in the Low-Temperature Phase. Acta Crystallogr. B 1981, 37 (11), 2101–2103. 10.1107/S056774088100811X.

[ref32] McCleverty J. A.; Meyer T. J. Comprehensive Coordination Chemistry II; Elsevier: Amsterdam, 2003. pp 15–513.

[ref33] Huber A.; Dubbert J.; Scherz T. D.; Voskuhl J. Design Concepts for Solution and Solid-State Emitters – A Modern Viewpoint on Classical and Non-Classical Approaches. Chem.—Eur. J. 2023, 29 (2), e202202481 10.1002/chem.202380261. 36193996 PMC10099667

[ref34] Terauchi H.; Sakai T.; Yamada Y. Ferroelasticity in Aniline-HBr. J. Phys. Soc. Jpn. 1980, 48 (1), 177–184. 10.1143/JPSJ.48.177.

[ref35] Liu Y.; Hu H.; Xu L.; Qiu B.; Liang J.; Ding F.; Wang K.; Chu M.; Zhang W.; Ma M.; Chen B.; Yang X.; Zhao Y. S. Orientation-Controlled 2D Anisotropic and Isotropic Photon Transport in Co-Crystal Polymorph Microplates. Angew. Chem., Int. Ed. 2020, 59 (11), 4456–4463. 10.1002/anie.201913441. 31889403

[ref36] Naumov P.; Sakurai K.; Ishikawa T.; Takahashi J.; Koshihara S.; Ohashi Y. Intramolecular Nitro-Assisted Proton Transfer in Photoirradiated 2-(2′,4′-Dinitrobenzyl)Pyridine: Polarized Optical Spectroscopic Study and Electronic Structure Calculations. J. Phys. Chem. A 2005, 109 (32), 7264–7275. 10.1021/jp0520392. 16834092

[ref37] Gaussian 16, Revision A.03, Frisch M. J.; Trucks G. W.; Schlegel H. B.; Scuseria G. E.; Robb M. A.; Cheeseman J. R.; Scalmani G.; Barone V.; Petersson G. A.; Nakatsuji H.; Li X.; Caricato M.; Marenich A. V.; Bloino J.; Janesko B. G.; Gomperts R.; Mennucci B.; Hratchian H. P.; Ortiz J. V.; Izmaylov A. F.; Sonnenberg J. L.; Williams-Young D.; Ding F.; Lipparini F.; Egidi F.; Goings J.; Peng B.; Petrone A.; Henderson T.; Ranasinghe D.; Zakrzewski V. G.; Gao J.; Rega N.; Zheng G.; Liang W.; Hada M.; Ehara M.; Toyota K.; Fukuda R.; Hasegawa J.; Ishida M.; Nakajima T.; Honda Y.; Kitao O.; Nakai H.; Vreven T.; Throssell K.; Montgomery J. A. Jr.; Peralta J. E.; Ogliaro F.; Bearpark M. J.; Heyd J. J.; Brothers E. N.; Kudin K. N.; Staroverov V. N.; Keith T. A.; Kobayashi R.; Normand J.; Raghavachari K.; Rendell A. P.; Burant J. C.; Iyengar S. S.; Tomasi J.; Cossi M.; Millam J. M.; Klene M.; Adamo C.; Cammi R.; Ochterski J. W.; Martin R. L.; Morokuma K.; Farkas O.; Foresman J. B.; Fox D. J. Gaussian, Inc.: Wallingford CT, 2016.

[ref38] Pang X.; Lv J.; Zhu C.; Qin L.; Yu Y. Photodeformable Azobenzene-Containing Liquid Crystal Polymers and Soft Actuators. Adv. Mater. 2019, 31 (52), 1904224 10.1002/adma.201904224. 31595576

[ref39] Yang Y.; Guan L.; Gao G. Low-Cost, Rapidly Responsive, Controllable, and Reversible Photochromic Hydrogel for Display and Storage. ACS Appl. Mater. Interfaces 2018, 10 (16), 13975–13984. 10.1021/acsami.8b00235. 29608057

[ref40] Wang X.; Xu B.; Tian W. Solid-State Luminescent Molecular Photoswitches. Acc. Mater. Res. 2023, 4 (4), 311–322. 10.1021/accountsmr.2c00158.

[ref41] Zhou X.; Xu X.; Zuo Y.; Liao M.; Shi X.; Chen C.; Xie S.; Zhou P.; Sun X.; Peng H. A Fiber-Shaped Light-Emitting Pressure Sensor for Visualized Dynamic Monitoring. J. Mater. Chem. C 2020, 8 (3), 935–942. 10.1039/C9TC05653J.

[ref42] Luo X.; Song W.; Gao F.; Shi J.; Cheng C.; Guo J.; He L.; Liu Q.; Yang Y.; Li S.; Wu Q. Strain-Modulated Light Emission Properties in a Single InGaN/GaN Multiple-Quantum-Well Microwire-Based Flexible Light-Emitting Diode. Adv. Eng. Mater. 2021, 23 (6), 2001430 10.1002/adem.202001430.

[ref43] Mutai T.; Sasaki T.; Sakamoto S.; Yoshikawa I.; Houjou H.; Takamizawa S. A Superelastochromic Crystal. Nat. Commun. 2020, 11 (1), 1824 10.1038/s41467-020-15663-5. 32286312 PMC7156499

[ref44] Zhang Z.; Xu N.; Huang Z.; Lai J.; Liu J.; Deng G.; Wang X.; Zhao W. High-Sensitivity Force Sensors Based on Novel Materials. Adv. Devices Instrum. 2023, 4, 0019 10.34133/adi.0019.

