# Supplementary material for: Ferroelastic Control of the Multicolor Emission from a Triply Doped Organic Crystal
Source: J Am Chem Soc. 2024 Jun 11;146(24):16540–8. doi: 10.1021/jacs.4c03190 (PMC11191679; doi:10.1021/jacs.4c03190)
Supplement: Supplementary file 1 — ja4c03190_si_001.pdf [file ja4c03190_si_001.pdf]

Legends for the Supplementary Movies

Supplementary Movie 1. A crystal of **APO@1** emitting light of different colors (orange, green, blue), depending on the excitation wavelength. The first image is from the crystal under white light.

Supplementary Movie 2. A pair of micromanipulators twinning and detwinning a crystal under polarized white light and UV light.

Supplementary Movie 3. Fluorescence recovery over time of each domain (parent, twinned, detwinned) monitored under excitation with 330–380 nm, 405 nm, and 488 nm light. The time is displayed in the upper right in hh:mm (hour:minute) format.

Supplementary Movie 4. Force/emission experiment measuring the color of light emitted in area 1 and area 2 of the crystal as it undergoes twin formation.
