# Supplementary material for: Ferroelastic Control of the Multicolor Emission from a Triply Doped Organic Crystal
Source: J Am Chem Soc. 2024 Jun 11;146(24):16540–8. doi: 10.1021/jacs.4c03190 (PMC11191679; doi:10.1021/jacs.4c03190)
Supplement: Supplementary file 6 — ja4c03190_si_006.pdf [file ja4c03190_si_006.pdf]

Supporting information

Ferroelastic Control of the Multicolor Emission from a Triply Doped Organic Crystal

Patrick Commins,[1○] Marieh B. Al-Handawi,[1○] Caner Deger,[2,3] Srujana Polavaram,[1] Ilhan Yavuz,[2] Rachid Rezgui,[1] Liang Li,[1,4]* K. N. Houk,[3] Panče Naumov[1,5,6,7]*

Affiliations:

[1]*Smart Materials Lab, New York University Abu Dhabi, PO Box 129188, Abu Dhabi, UAE*

[2]*Department of Physics, Marmara University, 34722 Istanbul, Türkiye*

[3]*Department of Chemistry and Biochemistry, University of California, Los Angeles, Los Angeles, CA, 90095-1569, USA*

[4]*Department of Sciences and Engineering, Sorbonne University Abu Dhabi, Abu Dhabi, PO Box 38044, Abu Dhabi, UAE*

[5]*Center for Smart Engineering Materials, New York University Abu Dhabi, Abu Dhabi, PO Box 129188, Abu Dhabi, UAE*

[6]*Research Center for Environment and Materials, Macedonian Academy of Sciences and Arts, Bul. Krste Misirkov 2, MK-1000 Skopje, Macedonia*

[7]*Molecular Design Institute Department of Chemistry, New York University, New York, NY 10003, USA*

[○]These authors contributed equally.

Materials and methods

Materials. Aniline was purchased from Sigma-Aldrich and distilled three times by vacuum distillation before use. All other reagents were purchased from commercial suppliers and used without further purification.

Crystallization of APO@1 and P@1 crystals. The crystals of **1** were grown by dissolving anthracene (3 mg), acridine orange (2 mg), aniline (0.9 mL) and 48% hydrobromic (0.45 mL) acid in methanol, (10 mL) and slowly evaporating the solution in a partially covered Erlenmeyer flask over 48 hours. The evaporation rate of 48 h is particularly important as phenazine is made *in situ* by the oxidation of aniline in air under acidic conditions, and if the solution evaporates too slowly phenazine will accumulate and become the most abundant guest in the crystal. Likewise, if the solution evaporates too quickly only trace amounts of phenazine will be formed. The resulting crystals were washed with acetone (10 mL, 6 times) until the acetone solution was colorless. The same protocol was used to create **P@1** crystals however, anthracene and acridine orange were not added into the crystallization solution.

The amount of dopant added to the crystals is the result of iterative crystallization with varying amounts of dopants. When loading amounts of anthracene and acridine are too high, the resulting crystals tend to be low-quality polycrystalline samples. Additionally, at a significantly high enough loading of anthracene, anthracene crystals can separate from the solution and contaminate the batch. When loadings of anthracene and acridine orange are too low, the emission of these compounds can be dwarfed by phenazine, which is formed *in situ*. The concentrations of 0.0025 ± .0001, 0.272 ± 0.163, and 0.0021 ± .0004% for A, P, and O, respectively, represent our optimized ratio of the emissive properties of dopants while maintaining a high crystal quality.

Optical microscopy. Optical microscopy images were acquired on a Nikon-LV Dia microscope using a Plan Fluor 5× objective. The images were captured using a *z*-stacking algorithm by acquiring images of the crystal at regular *z* intervals and performing a *z*-stacking function to construct a cumulative image.

Fluorescence recovery. The fluorescence recovery experiments were performed by modifying the Nikon-LV-Dia microscope with a high-pressure Hg lamp (Nikon Intensilight C-HGFIE) and using two UV filters for the incident light: one filter that blocks light below 330 nm, and another that blocks light above 380 nm. The setup was also used to acquire the ex. 330–380 nm polar plot and for the cyclability experiments. The polar plot was acquired by twinning and detwinning the crystal and allowing it to rest for 100 min. Afterwards the crystal was imaged at approximately 10º intervals by rotating the stage and acquiring images. The fluorescence intensity of the respective domains at each rotation were measured using the software Fiji.[1]

Photomechanical force sensing cycling experiments. Fluorescence images of pristine crystals were captured using the modified Nikon-LV-Dia microscope under UV irradiation. The crystals were twinned, and then detwinned at 25 °C. Then they were left in the dark for 30 min, and fluorescence images were acquired. The experiment was repeated for 5 cycles using the same crystal and detwinned in the same region.

Thermal recovery of emission in detwinned domain. A single crystal of **APO@1** was twinned and detwinned at multiple locations. Images of the crystal were captured every 10 mins for a duration of 120 minutes. Each detwinned region was exposed to different temperatures 25, 50 and 70 °C using a hot stage Fluorescent images of the crystals were captured using the modified the Nikon-LV-Dia microscope under UV irradiation.

Fluorescence spectroscopy. Fluorescence emission experiments were performed using a Jasco FP8500 spectrofluorometer with 1 cm path length with 3 mL quartz cuvettes. The solvents used for the UV-Vis and emission spectroscopy were of analytical grade and purchased from Sigma-Aldrich.

CIE color plot. The 1931 CIE color plot was constructed by acquiring excitation/emission spectra of an **APO@1** powder from 340–540 nm in 10 nm intervals using a Jasco FP8500 spectrofluorometer. The emission spectra were then converted into its corresponding CIE color coordinates using the Jasco's Color analysis software.[2]

Confocal fluorescence microscopy. Confocal optical microscopy images and polar plots were created using two microscopes. The first was an upright confocal/two-photon Olympus FluoView FV1000MPE microscope with an upright Olympus UPlanFL N 10×/0.30 NA objective. The laser line was 405 nm and 488 nm and had a spectral detection range of 415–455 and 495–525 nm, respectively. The second microscope was an inverted confocal/2-Photon Microscope Olympus FluoView FV1000MPE with a HC PL APO CS2 10×/0.40 DRY objective. The laser line was 405 nm and 488 nm and had a spectral range of 416–481 nm and 495–582 nm, respectively.

Fluorescence lifetime measurements. Fluorescence lifetime measures were acquired using an Super Resolution Microscope - Leica STED 3X. In this experiment, confocal fluorescence lifetime microscopy was employed with a logical size of 256 pixels along the X and Y dimensions, each spanning 1,550 µm, and a pixel size of 6.078 µm. The temporal aspect was captured across 100 frames, with a total duration of 1 minute and 54.477 seconds. The imaging was conducted using a HC PL FLUOTAR 10x/0.30 DRY objective with a numerical aperture of 0.3 and a pixel dwell time of 2.425 µs. Laser sources included a 405 nm laser with pulsed output. The experiment utilized a HyD detector with 415-793 nm detection range.

The amount of Förster resonance energy transfer (FRET) between phenazine and acridine orange was calculated by measuring the lifetime of phenazine in an anilinium bromide crystal doped with only phenazine (**P@1**) vs the lifetime of phenazine in **APO@1**. The % energy transfer was calculated using the following equation:

$$Energy\ transfer = \left(1 - \frac{\tau_{DA}}{\tau_D}\right) * 100\%$$

Where t_{DA} is the lifetime of the donor in presence of the acceptor and t_D is the lifetime of the donor alone. A 100 lifetimes were measured for crystals of **P@1** and **APO@1** each and results are shown in Supplementary Tables 1 and 2. The results show that the lifetime of **P@1** is 1.93 ns and the lifetime of **APO@1** is 1.74 ns, which gives a % energy transfer of 9.8% of phenazine to acridine orange.

Polar plots. Polar plots at excitation of 405 and 488 nm were acquired on the Olympus FluoView FV1000MPE microscope with an Olympus UPlanFL N 10×/0.30 NA objective. The crystal was detwinned and allowed to rest for 100 min. Afterwards the crystal was imaged at approximately 10° intervals by rotating the stage and acquiring images. The fluorescence intensity of the respective domains at each rotation were measured using Fiji.[1]

Computational methods. Density functional theory (DFT) is used to perform quantum mechanical calculations for the ground and excited states as implemented in Gaussian 16.[2] B3LYP/6-311G(d,p) level of theory is used to optimize the geometries. UV/Vis absorption spectra were calculated based on time-dependent density functional theory (TDDFT).[3]

Tensile testing and force luminosity plot. Tensile measurements were performed on MTII/Fullam Semtester tensile tester (MTI Instruments) using a 5 lbs load cell. then mounted onto the tensile tester using a small amount of UV curable resin (Qiao Qiao UV Resin Hard). The crystals were pulled and compressed in a sinusoidal manner at an amplitude of 0.015 mm and 160 s per cycle. While undergoing the sinusoidal tension-compression cycle, the crystal was irradiated with polarized 350–380 nm light and the emission was captured using a Dino-lite AM7115MT-FUW camera. The video was processed using Fiji[1] to separate the RGB components of the emitted light and measured the intensity of each color over time.

Mass spectrometry. All LC-MS and analyses were performed on an Agilent 6540 UHD Accurate-Mass Quadrupole – Time of Flight spectrometer using a multimode (ESI and APCI) source. The LC system consisted of an Agilent 1290 Infinity II UHPLC. The MS QTOF was operated at a *m*/*z* range of 100–1000 amu, with fragmentor set to 175 V, and a skimmer at 65 V. The source was set to: gas temperature 300 °C, vaporizer 200 °C, drying gas 8 L min^{-1}, nebulizer 35 psi. The mass spectra were acquired and processed with the Masshunter software.[4]

X-ray diffraction data. The X-ray crystal structure data on anilinium bromide was accessed from the Cambridge Crystallographic Data Centre (CCDC) deposition number 299874 and 2043129. To confirm the structure, X-ray diffraction data was collected using Bruker Apex III single crystal diffractometer. The integration and scaling of the data were performed by SAINT.[5] The diffraction data were corrected for absorption effects by SADABS.[6] The structures were solved and refined using the Olex2 program.[7] The non-hydrogen atoms were refined anisotropically. The positions of hydrogen atoms were calculated and refined isotropically. The twinned structure was confirmed by localized face indexing and data collection on selected regions of the crystal.

Powder X-ray diffraction. Powder X-ray diffraction experiments were performed on a Panalytical Empyrean using copper as a source (1.5418 Å) at power settings of 45 kV and 40 mA. The beam passed through a slit that illuminated the powder sample at 0.5°. The diffracted beam was measured using a Pixcel solid-state detector with a beta filter and a resolution of 0.0134°. The material was ground by hand using a mortar and pestle for a set duration after which the diffraction pattern was immediately acquired.

Supporting References

1. Schindelin, J.; Arganda-Carreras, I.; Frise, E.; Kaynig, V.; Longair, M.; Pietzsch, T.; Preibisch, S.; Rueden, C.; Saalfeld, S.; Schmid, B.; Tinevez, J.-Y.; White, D. J.; Hartenstein, V.; Eliceiri, K.; Tomancak, P.; Cardona, A. Fiji: An Open-Source Platform for Biological-Image Analysis. *Nat. Methods* **2012**, *9* (7), 676–682. https://doi.org/10.1038/nmeth.2019.
2. 2. Jasco Spectra Manager Version 2, Luminous Color Analysis software, version 2.4.1.2.
3. Gaussian 16, Revision A.03, M. J. Frisch, G. W. Trucks, H. B. Schlegel, G. E. Scuseria, M. A. Robb, J. R. Cheeseman, G. Scalmani, V. Barone, G. A. Petersson, H. Nakatsuji, X. Li, M. Caricato, A. V. Marenich, J. Bloino, B. G. Janesko, R. Gomperts, B. Mennucci, H. P. Hratchian, J. V. Ortiz, A. F. Izmaylov, J. L. Sonnenberg, D. Williams-Young, F. Ding, F. Lipparini, F. Egidi, J. Goings, B. Peng, A. Petrone, T. Henderson, D. Ranasinghe, V. G. Zakrzewski, J. Gao, N. Rega, G. Zheng, W. Liang, M. Hada, M. Ehara, K. Toyota, R. Fukuda, J. Hasegawa, M. Ishida, T. Nakajima, Y. Honda, O. Kitao, H. Nakai, T. Vreven, K. Throssell, J. A. Montgomery, Jr., J. E. Peralta, F. Ogliaro, M. J. Bearpark, J. J. Heyd, E. N. Brothers, K. N. Kudin, V. N. Staroverov, T. A. Keith, R. Kobayashi, J. Normand, K. Raghavachari, A. P. Rendell, J. C. Burant, S. S. Iyengar, J. Tomasi, M. Cossi, J. M. Millam, M. Klene, C. Adamo, R. Cammi, J. W. Ochterski, R. L. Martin, K. Morokuma, O. Farkas, J. B. Foresman, and D. J. Fox, Gaussian, Inc., Wallingford CT, 2016.
4. Agilent MassHunter Workstation Qualitative Analysis, Version 10.0.
5. SAINT, Bruker AXS, Inc., Madison, WI (2006).
6. SADABS, Bruker AXS, Inc., Madison, WI (2006).
7. Dolomanov, O. V.; Bourhis, L. J.; Gildea, R. J.; Howard, J. a. K.; Puschmann, H. OLEX2: A Complete Structure Solution, Refinement and Analysis Program. *J. Appl. Cryst.* **2009**, *42* (2), 339–341. https://doi.org/10.1107/S0021889808042726.

Supporting Figures

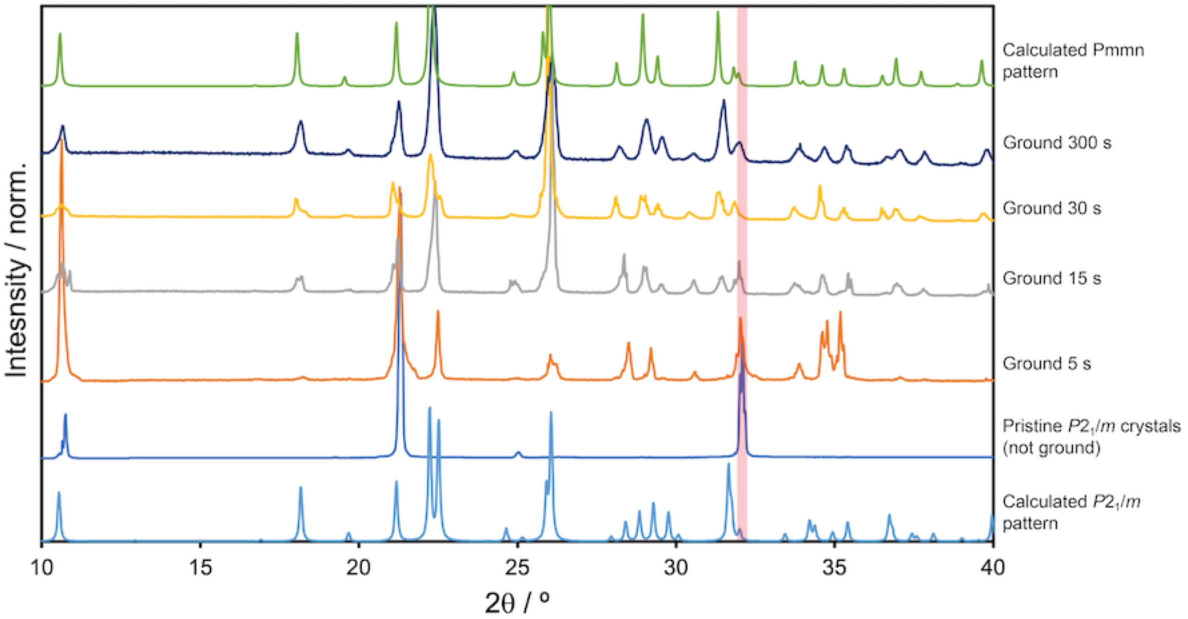

Figure S1. A series of PXRD diffractograms showing the grinding-induced phase transition of the **APO@1** crystals from the $P2_1/m$ phase to the *Pmmn* phase. Performing PXRD on unground crystals leads to preferential orientation and only the (002), (004) and (006) are found at 11, 21, and 32°, respectively and many of the other peaks are not observed. However, even with the limited information the two phases are distinguishable and the unground crystals are the $P2_1/m$ phase. When the sample is ground peaks from the *Pmmn* phase become visible and the (006) peak at 32° from the $P2_1/m$ is no longer present. The red shaded area highlights the (006) peak at 32° that distinguishes the $P2_1/m$ phase from the *Pmmn* phase. The crystals were ground by hand in a mortar and pestle.

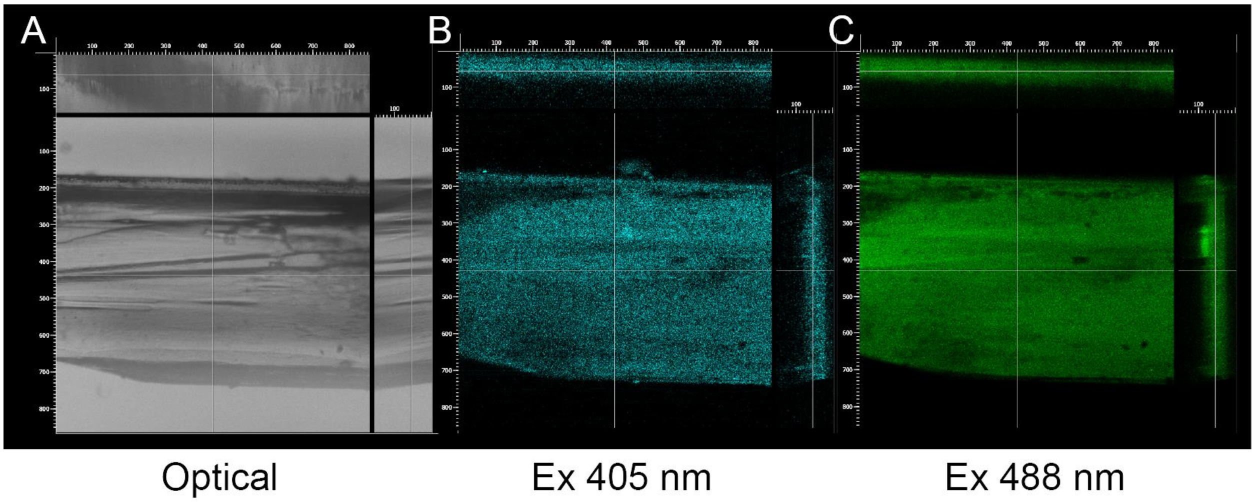

Figure S2. *Z*-stacked confocal fluorescence images of an **APO@1** crystal excited at 405 and 488 nm. The side panels above and to the right of the center images are line scans of the fluorescence intensity for the x and y directions at varying *z* heights. Each line scan shows approximately 70 mm of depth into the crystal. There is a nearly continuous fluorescence intensity through the crystals in the *x*, *y*, and *z* directions.

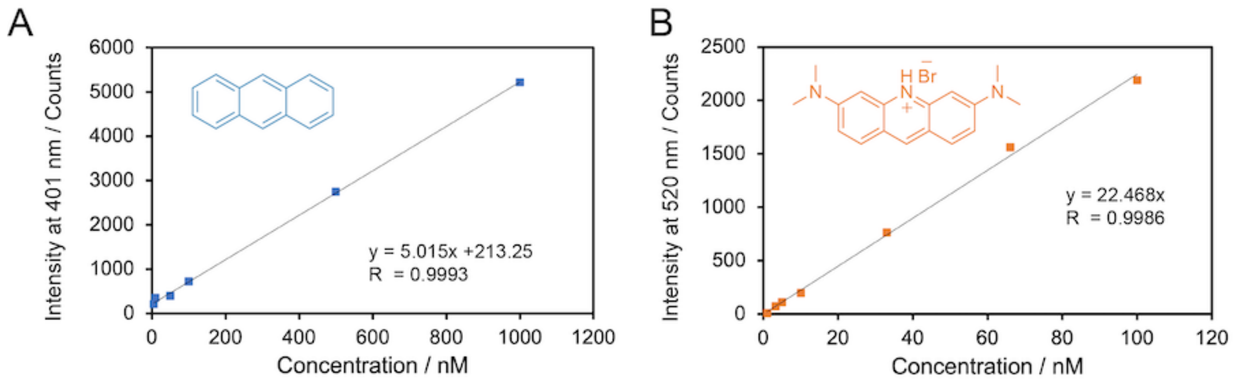

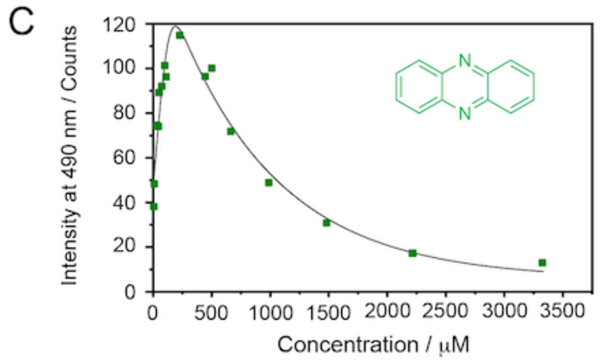

Model	GaussMod
Equation	double z = (x-xc)/w - w/t0; y = y0 + A/t0 * exp(0.5*(w/t0)^2-(x-xc)/t0)*(erf(z/sqrt(2))+1)/2
Plot	B
y0	-4.25505 ± 8.30921
A	122838.58923 ± 27438.46881
xc	38.33534 ± 13.35171
w	83.58654 ± 13.48295
t0	878.71564 ± 198.7019
Reduced Chi-Sqr	55.50352
R-Square (COD)	0.961
Adj. R-Square	0.94682

Figure S3. Fitted fluorescence emission for **A** (A), **P**(C) and **O** (B) at various concentrations in ethanol. Compound **A**, **P** and **O** were excited at 350, 390, and 480 nm, respectively. A known mass of **APO@1** crystals were dissolved in ethanol and their respective emission intensities were measured. The concentration of the emitter in the **APO@1** sample was then determined from the emission intensity at the specified wavelengths of 401 nm, 490 and 520 nm for **A**, **P**, and **O**, respectively. The data for **A** and **O** was fit using a linear relationship between concentration and intensity. The data for **P** showed signs of aggregation-induced quenching, and was fit using a modified Gaussian. The guest concentrations were found to be 496 ± 16 nM, 56.9 ± 34.2 µM, and 281 ± 62 nM or 0.0025 ± .0001, 0.272 ± 0.163, 0.0021 ± .0004% of the mass of the **APO@1** crystals (*m*/*m*) for **A**, **P** and **O**, respectively

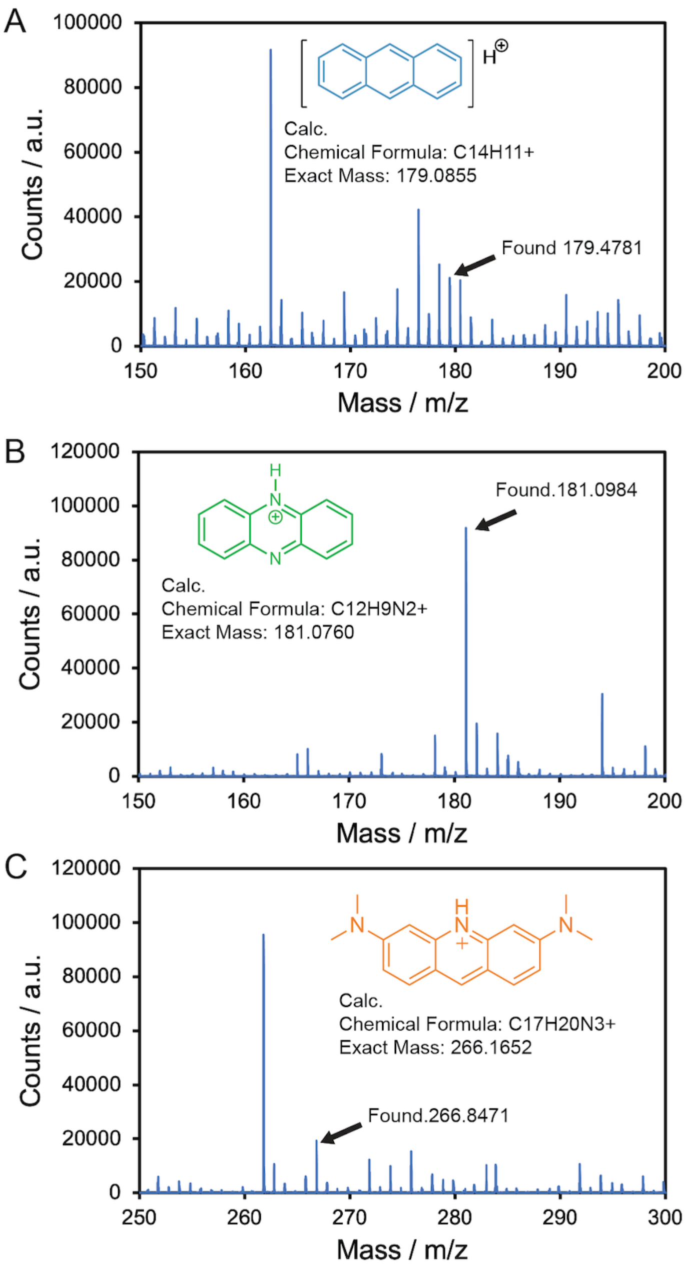

Figure S4. Mass spectroscopy of **A**, **P** and **O** found by extraction from **APO@1** by trituration with dichloromethane for **A** and **O** and benzene for **P**. **A** was ionized using atmospheric pressure chemical ionization (APCI), and **P** and **O** were ionized using electrospray ionization (ESI).

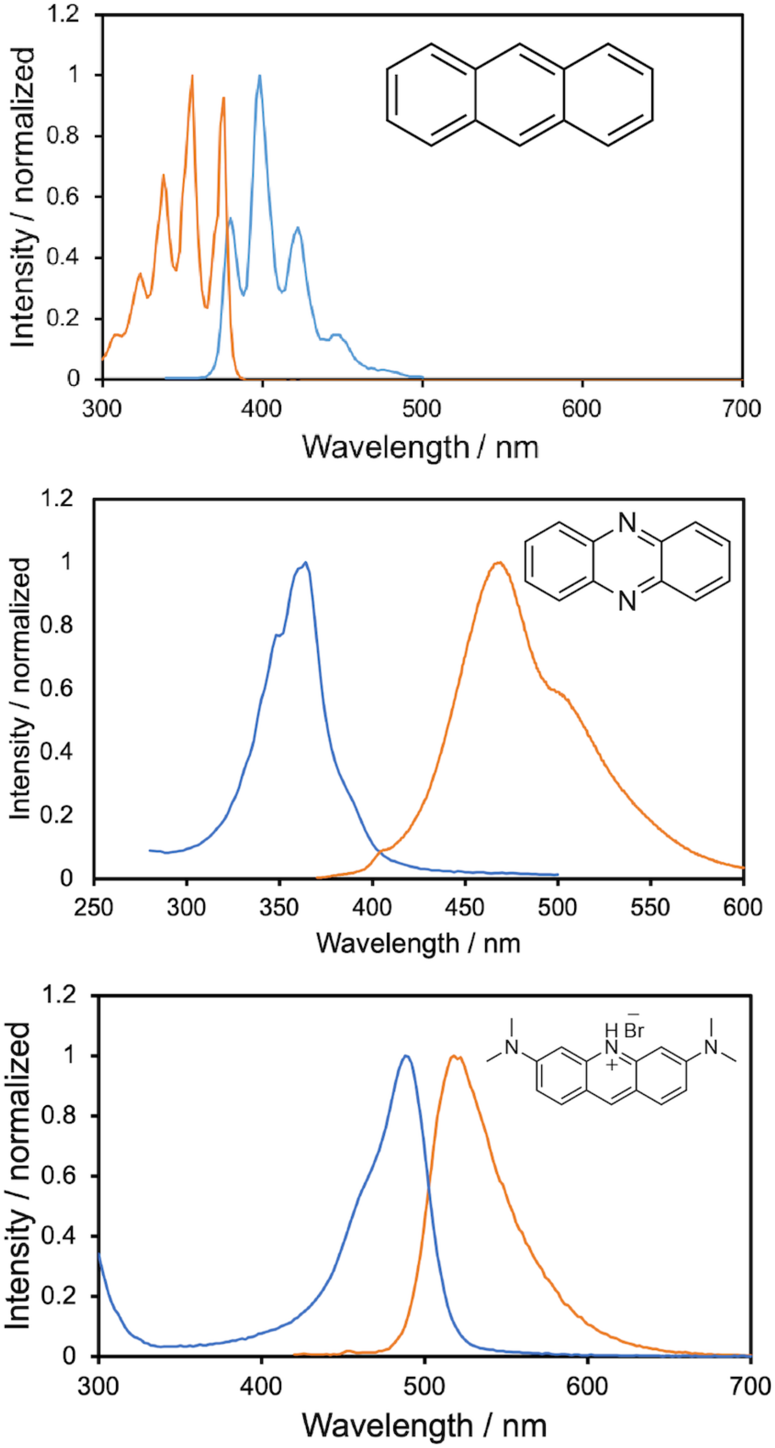

Figure S5. Absorption and emission spectra of **A**, **P**, and **O** in cyclohexane, dichloromethane and ethanol, respectively. The molar absorptivity constants for **A**, **P**, and **O** are 9700, 16,900 and 27,000 M^{-1} cm^{-1} in cyclohexane, dichloromethane and ethanol, respectively.

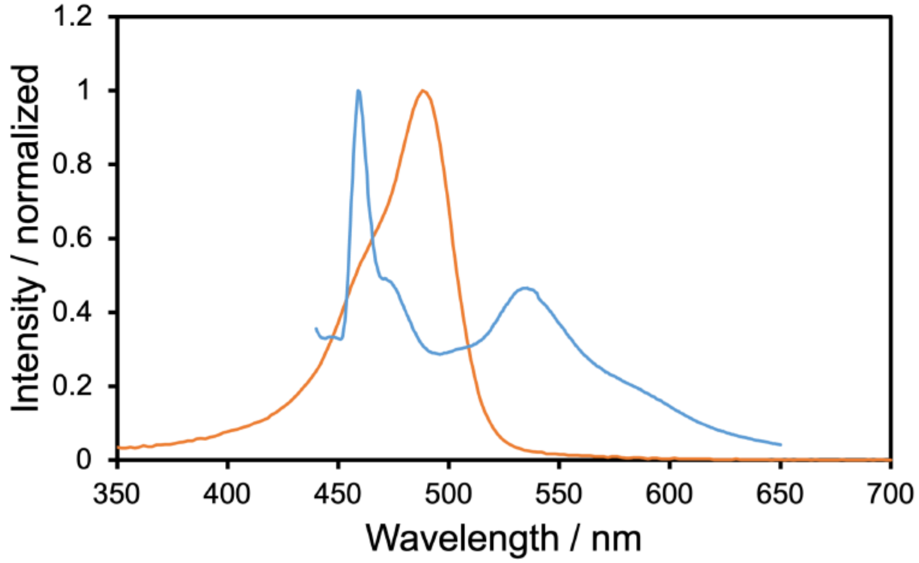

Figure S6. The UV-Vis absorption spectrum of acridine orange in MeOH (orange) compared against the emission spectrum of **APO@1** (blue) powder excited at 420 nm.

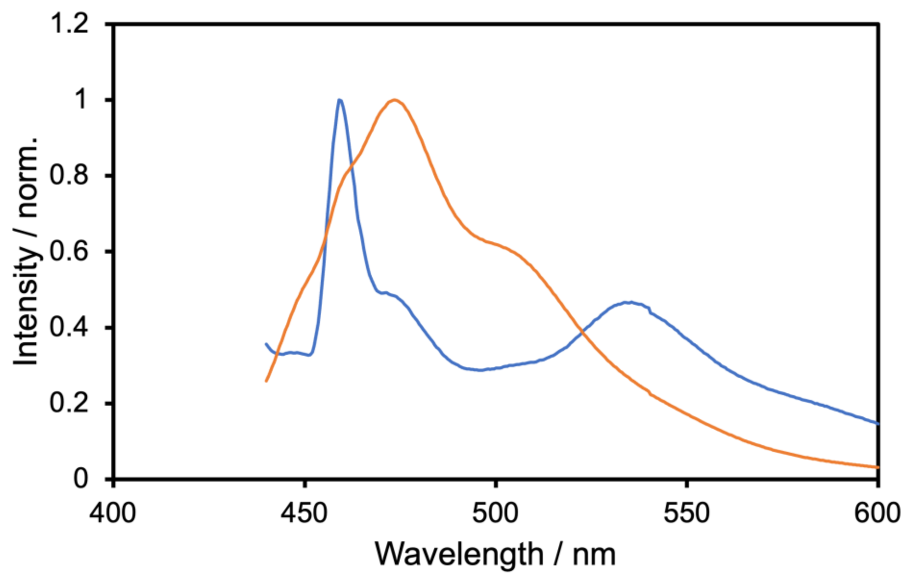

Figure S7. Emission spectrum of **APO@1** (blue) and **P@1** (orange) powders excited at 420 nm. **P@1** has a maximum at 471 nm and two shoulders at 460 and 506 nm. **APO@1** has a maximum at 460 nm and a shoulder at 471 nm, indicating the higher wavelength emissions are being transferred to acridine orange.

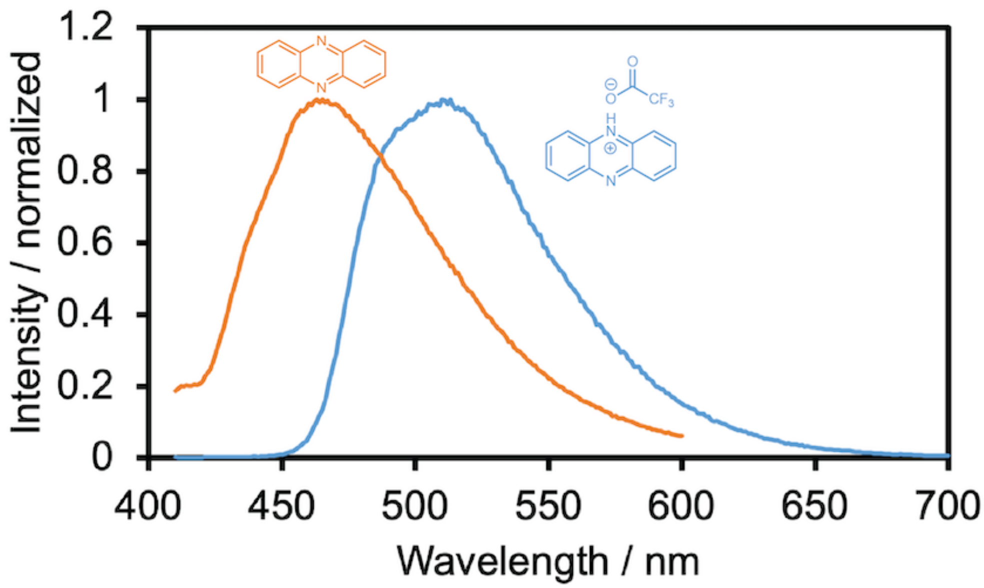

Figure S8. Emission spectra of **P** excited at 360 nm in methanol (orange) and of **P** with a drop of trifluoroacetic acid in methanol (blue). The spectrum of phenazinium cation is notably red-shifted, with a maximum at 517 nm.

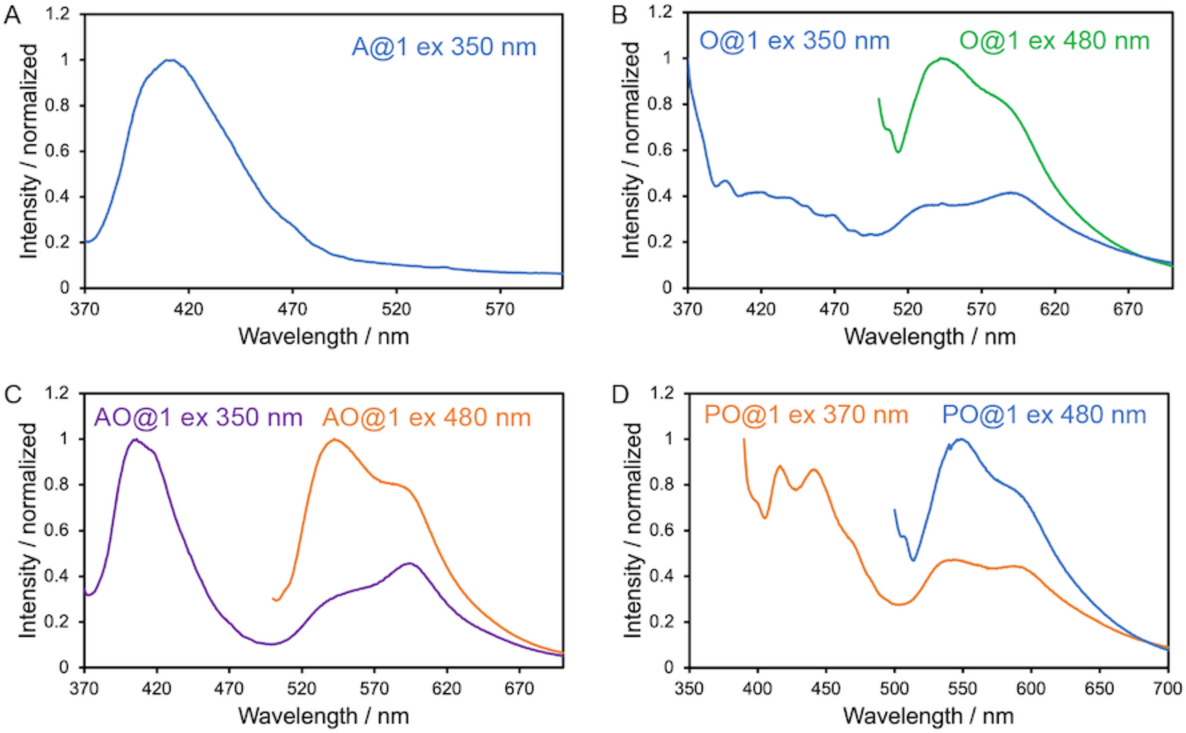

Figure S9. The emission spectra for **A@1**, **O@1**, **AO@1** and **PO@1** excited at their respective absorption maximum wavelengths.

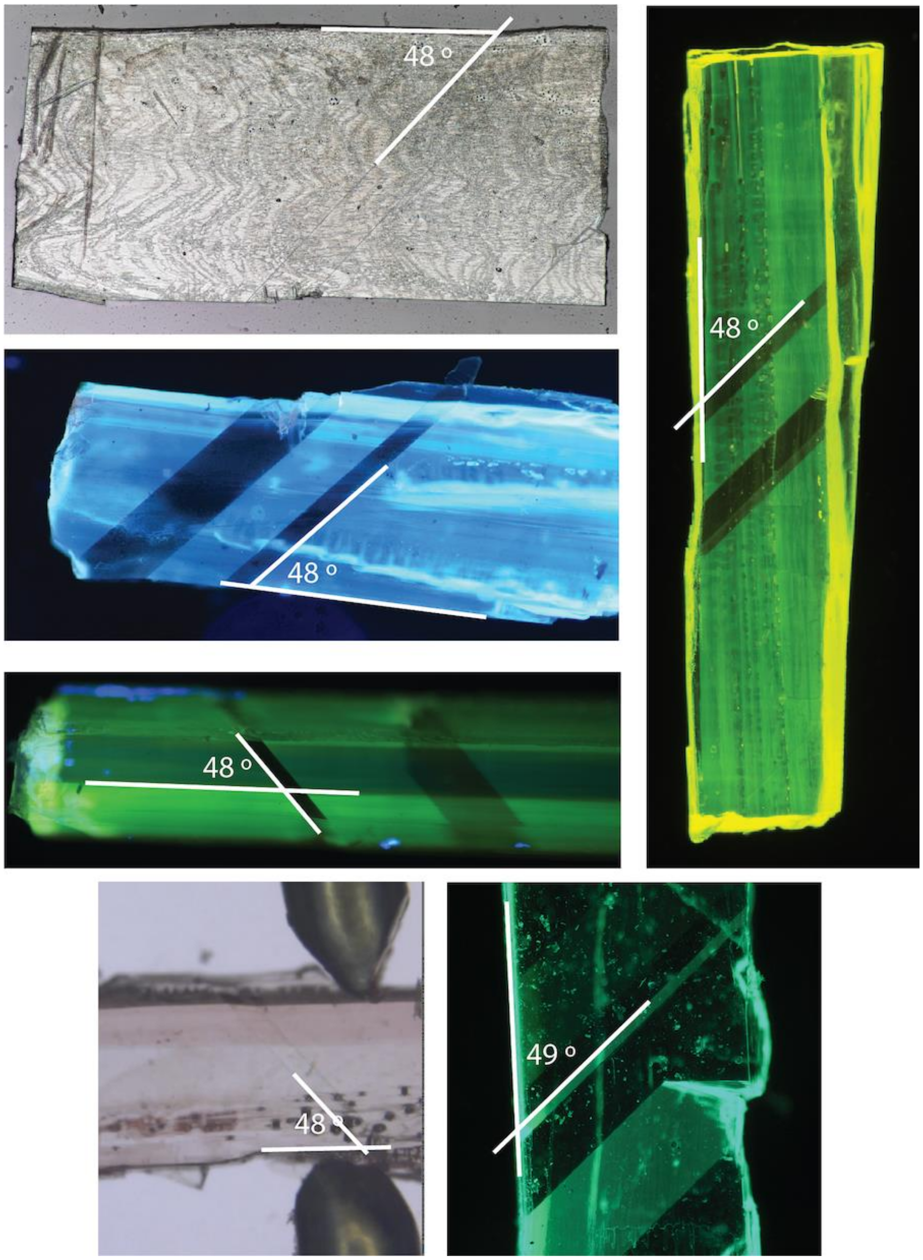

Figure S10. Polarized optical microscopy images of twinned and detwinned crystals of **APO@1** showing the measurement of the angle of the twin domain.

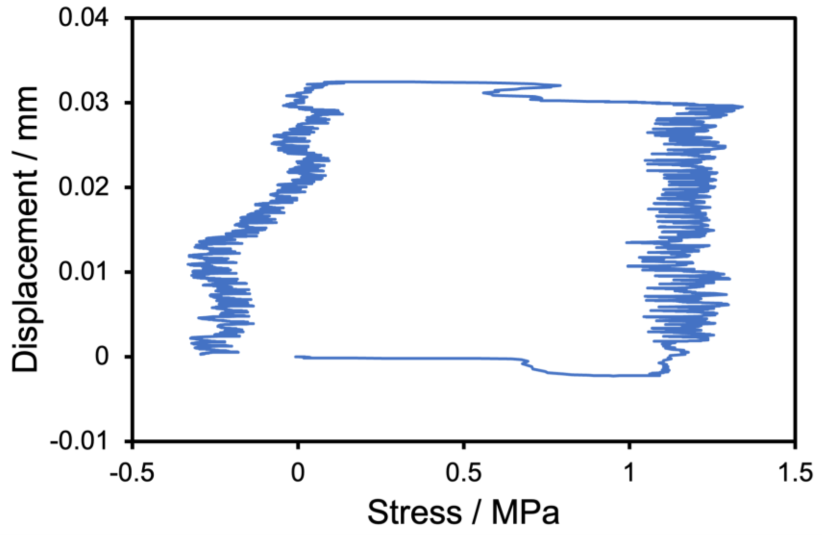

Figure S11. A stress displacement curve showing the ferroelastic effect in a crystal of **APO@1**. When a tensile load is applied to the crystal, a sudden increase in stress is measured to induce the twin transition. Once the twin transformation is initiated, constant stress is necessitated to propagate the transition. After 0.03 mm of displacement, an equal amount of compressive load is required to begin the detwinning. Once the detwinning is initiated, constant stress is necessary to return the crystal back to its parent phase.

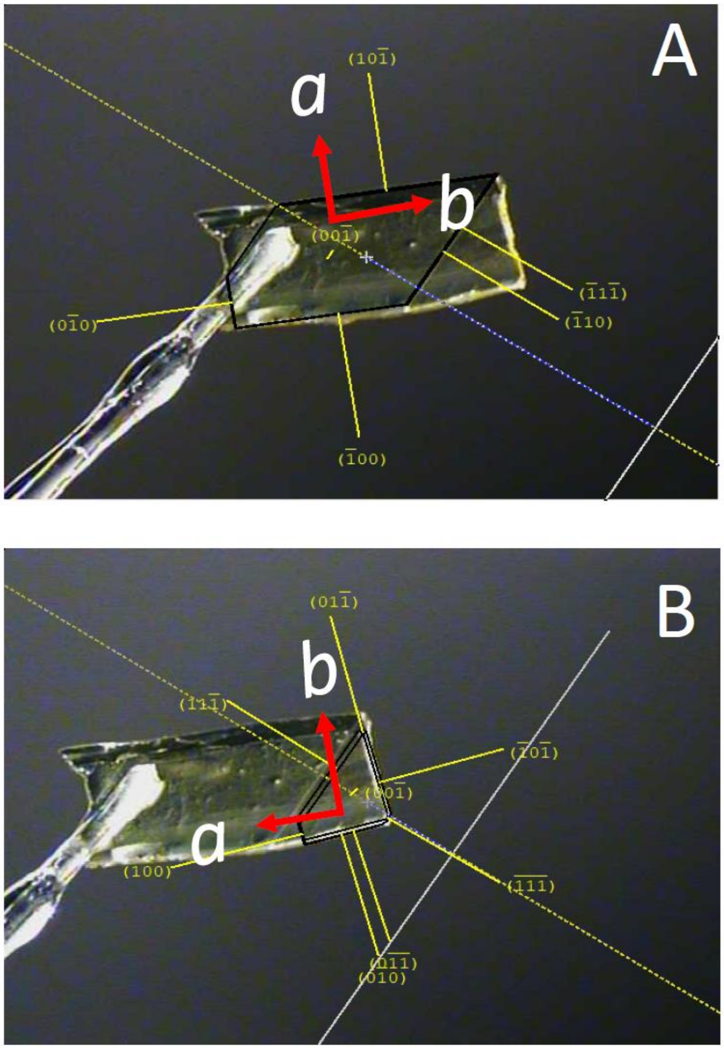

Figure S12. Face indexing, based on X-ray diffraction, confirming that the parent domain (A) coexists with the twin domain (B).

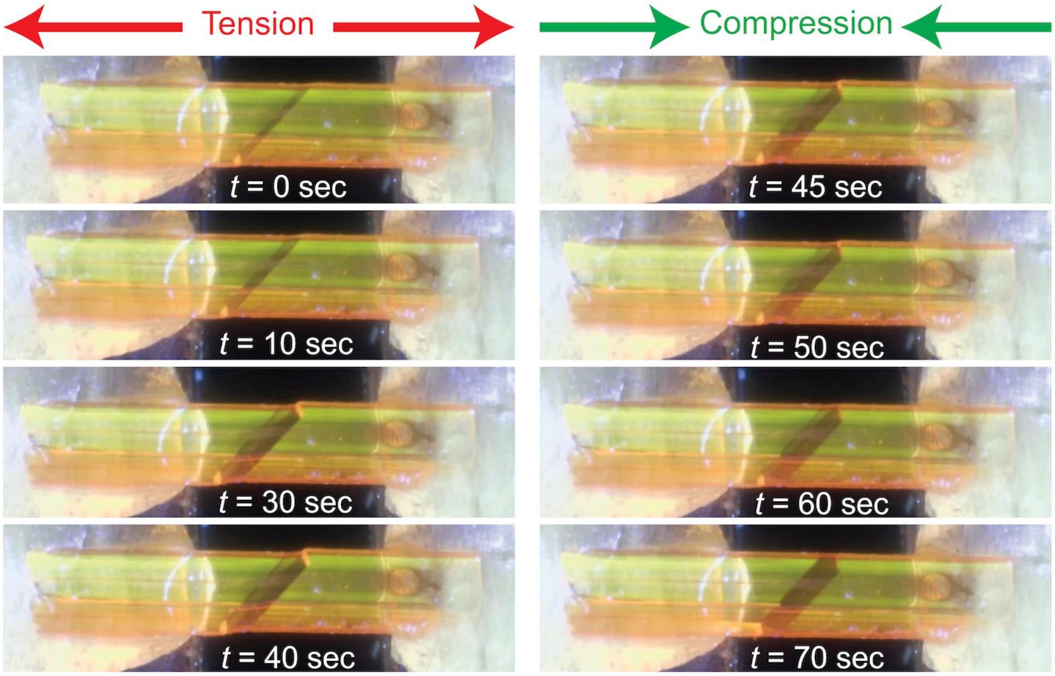

Figure S13. Fluorescence images of a single crystal under a tensile load, revealing the ferroelastic twin and changes in the fluorescence intensity in the twin domain.

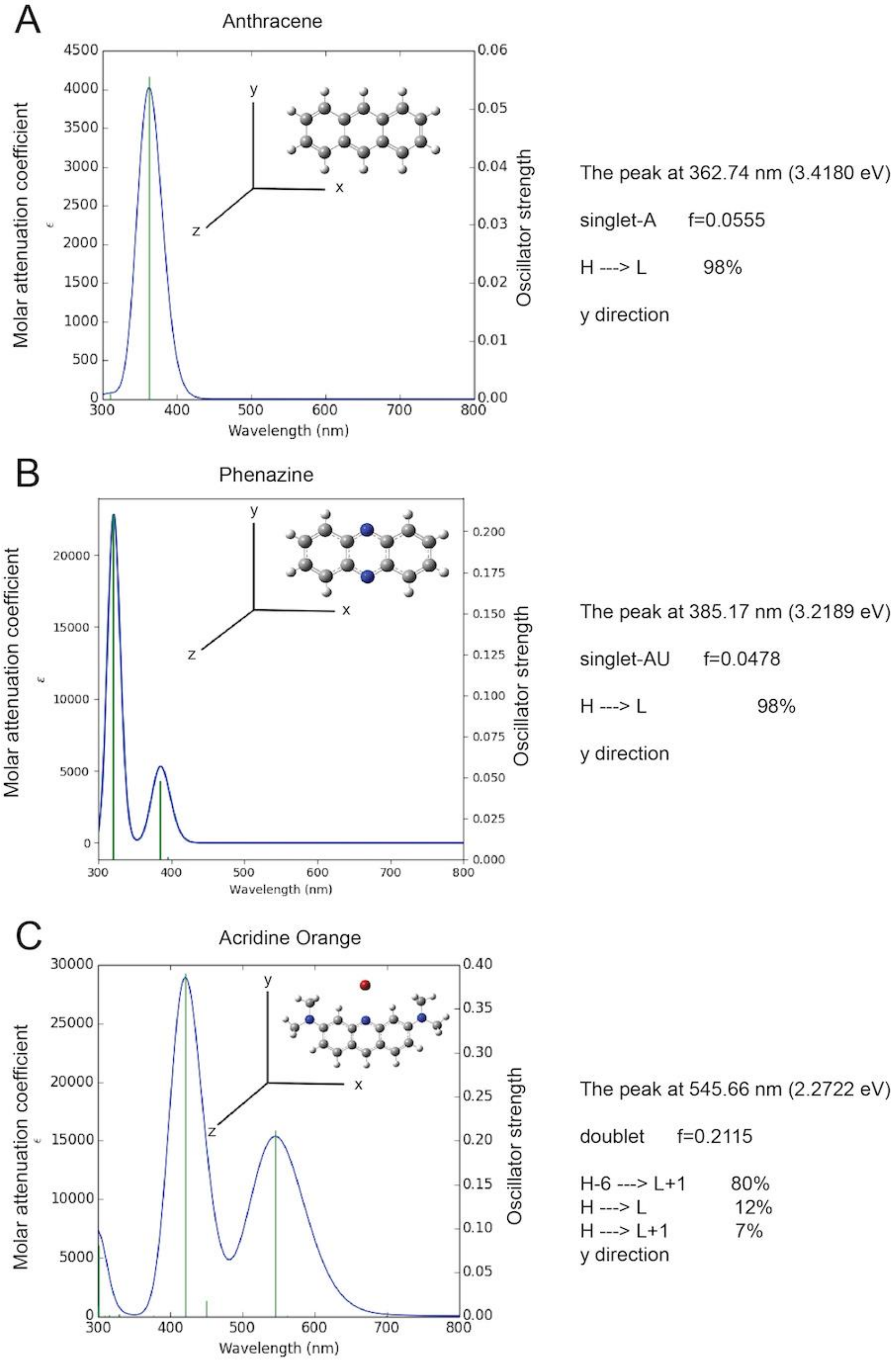

Figure S14. DFT-calculated UV-Vis spectra and oscillator strengths of anthracene (A), phenazine (B), and acridine orange (C). The origins of excitations and their contributions are given on the right panel.

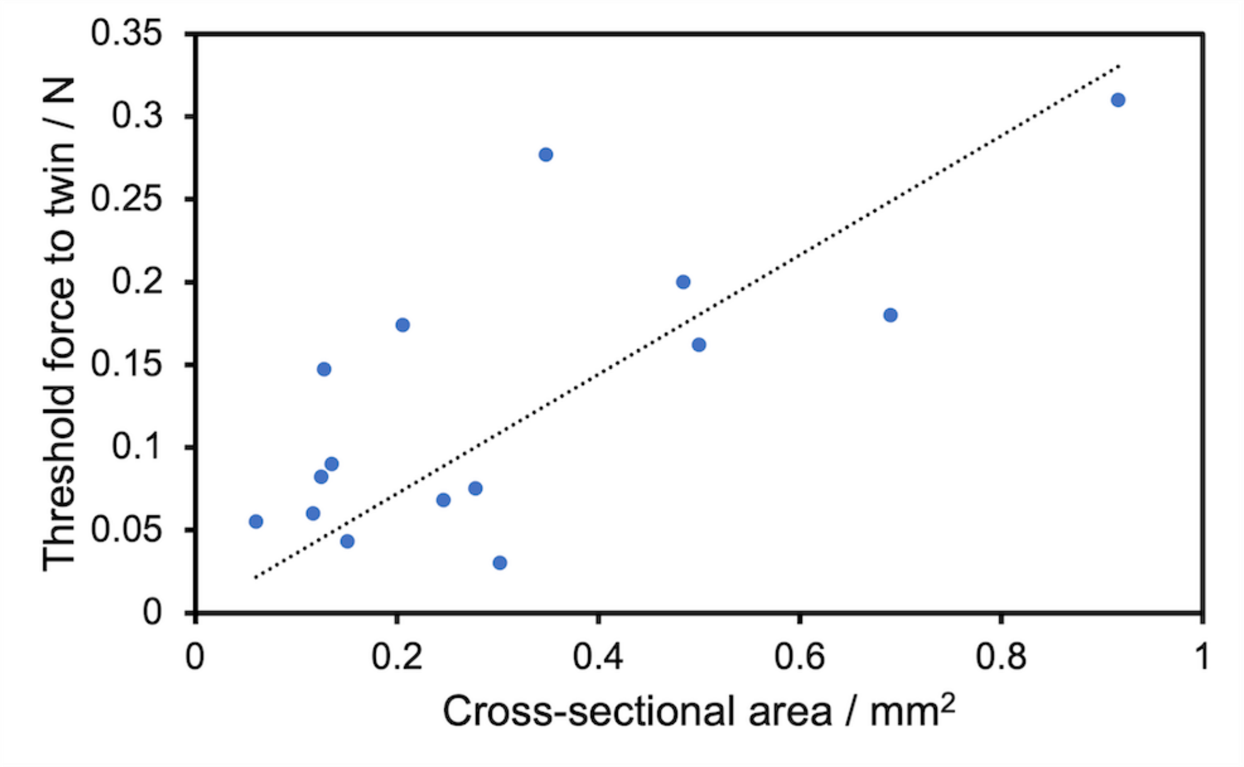

Figure S15. A comparison of the threshold force required to twin a crystal of **APO@1** and the cross-sectional area of the crystal (*n* = 15). The plot shows that larger crystals with higher cross-sectional area, require more force than smaller crystals to induce a twin domain. A trendline is added to guide the eye.

Supporting Tables

Table S1. Lifetimes of phenazine in **P@1** in the solid with excitation at 405 nm

Number	Lifetime (τ) 1 ns	Lifetime (τ) 2 ns	Mean τ, Intensity Weighted ns
1	1.056	3.171	1.927
2	1.046	3.181	1.915
3	1.057	3.193	1.914
4	1.079	3.226	1.919
5	1.085	3.248	1.926
6	1.086	3.231	1.920
7	1.090	3.270	1.924
8	1.094	3.270	1.926
9	1.097	3.276	1.926
10	1.112	3.335	1.937
11	1.109	3.281	1.928
12	1.103	3.293	1.930
13	1.105	3.308	1.929
14	1.115	3.308	1.932
15	1.116	3.329	1.935
16	1.123	3.351	1.934
17	1.124	3.341	1.935
18	1.119	3.313	1.930
19	1.124	3.320	1.932
20	1.126	3.342	1.935
21	1.128	3.354	1.936
22	1.124	3.325	1.928
23	1.134	3.359	1.940
24	1.119	3.311	1.931
25	1.135	3.377	1.941
26	1.131	3.354	1.935
27	1.123	3.320	1.928
28	1.133	3.368	1.935
29	1.135	3.382	1.940
30	1.138	3.376	1.937
31	1.141	3.407	1.938
32	1.126	3.327	1.931
33	1.135	3.373	1.938
34	1.132	3.348	1.935
35	1.129	3.362	1.939
36	1.127	3.328	1.931
37	1.126	3.313	1.931
38	1.131	3.347	1.934
39	1.138	3.349	1.934
40	1.144	3.400	1.938
41	1.129	3.335	1.935

42	1.133	3.331	1.933
43	1.148	3.395	1.939
44	1.137	3.340	1.932
45	1.136	3.353	1.936
46	1.121	3.306	1.926
47	1.137	3.354	1.937
48	1.143	3.380	1.939
49	1.135	3.349	1.930
50	1.134	3.358	1.933
51	1.134	3.348	1.933
52	1.134	3.338	1.932
53	1.133	3.342	1.935
54	1.135	3.373	1.939
55	1.141	3.377	1.937
56	1.136	3.349	1.932
57	1.135	3.376	1.937
58	1.145	3.395	1.937
59	1.136	3.343	1.933
60	1.150	3.398	1.943
61	1.148	3.411	1.943
62	1.147	3.387	1.938
63	1.152	3.411	1.945
64	1.150	3.395	1.937
65	1.144	3.382	1.935
66	1.142	3.379	1.938
67	1.142	3.359	1.935
68	1.146	3.396	1.939
69	1.153	3.420	1.942
70	1.140	3.381	1.938
71	1.152	3.396	1.939
72	1.149	3.388	1.936
73	1.144	3.394	1.940
74	1.143	3.394	1.936
75	1.148	3.404	1.939
76	1.149	3.403	1.942
77	1.143	3.378	1.939
78	1.140	3.365	1.932
79	1.147	3.407	1.944
80	1.142	3.367	1.934
81	1.156	3.414	1.942
82	1.148	3.402	1.935
83	1.138	3.346	1.933
84	1.150	3.390	1.940
85	1.154	3.396	1.937
86	1.147	3.402	1.941
87	1.147	3.385	1.936
88	1.150	3.400	1.937

89	1.146	3.380	1.935
90	1.142	3.389	1.940
91	1.148	3.408	1.939
92	1.162	3.455	1.948
93	1.147	3.394	1.937
94	1.142	3.389	1.937
95	1.158	3.419	1.941
96	1.151	3.400	1.939
97	1.151	3.374	1.934
98	1.137	3.358	1.933
99	1.143	3.393	1.937
100	1.152	3.405	1.941
**Average**	1.132	3.355	1.935
**Std Dev**	0.022	0.052	0.006

Table S2. Lifetimes of phenazine in **APO@1** in the solid with excitation at 405 nm

Number	Lifetime (τ) 1 ns	Lifetime (τ) 2 ns	Mean τ, Intensity Weighted ns
1	1.114	2.226	1.718
2	1.220	2.344	1.732
3	1.254	2.374	1.733
4	1.256	2.385	1.735
5	1.268	2.401	1.735
6	1.245	2.374	1.735
7	1.265	2.391	1.736
8	1.289	2.440	1.738
9	1.254	2.381	1.735
10	1.286	2.438	1.739
11	1.278	2.423	1.737
12	1.295	2.454	1.741
13	1.285	2.428	1.737
14	1.271	2.391	1.738
15	1.284	2.417	1.737
16	1.293	2.443	1.737
17	1.256	2.373	1.734
18	1.264	2.382	1.736
19	1.276	2.416	1.737
20	1.283	2.436	1.740
21	1.278	2.405	1.738
22	1.299	2.459	1.740
23	1.283	2.425	1.738
24	1.293	2.437	1.738
25	1.279	2.405	1.737
26	1.282	2.420	1.739

27	1.298	2.456	1.741
28	1.281	2.419	1.739
29	1.287	2.418	1.738
30	1.286	2.408	1.741
31	1.299	2.436	1.738
32	1.284	2.422	1.740
33	1.309	2.462	1.741
34	1.295	2.431	1.739
35	1.293	2.438	1.739
36	1.294	2.438	1.740
37	1.297	2.450	1.741
38	1.293	2.437	1.740
39	1.313	2.472	1.742
40	1.294	2.442	1.741
41	1.308	2.458	1.741
42	1.311	2.480	1.742
43	1.300	2.456	1.739
44	1.322	2.486	1.744
45	1.289	2.437	1.740
46	1.305	2.462	1.741
47	1.308	2.466	1.742
48	1.317	2.487	1.743
49	1.316	2.482	1.744
50	1.316	2.479	1.743
51	1.316	2.484	1.743
52	1.320	2.478	1.742
53	1.292	2.423	1.740
54	1.301	2.438	1.740
55	1.312	2.459	1.743
56	1.298	2.432	1.740
57	1.304	2.441	1.743
58	1.320	2.475	1.744
59	1.305	2.445	1.741
60	1.319	2.483	1.744
61	1.302	2.441	1.740
62	1.316	2.464	1.743
63	1.304	2.456	1.743
64	1.319	2.481	1.743
65	1.305	2.441	1.741
66	1.322	2.483	1.744
67	1.309	2.463	1.741
68	1.302	2.440	1.741
69	1.331	2.511	1.746
70	1.310	2.464	1.739
71	1.287	2.411	1.740
72	1.326	2.502	1.745
73	1.312	2.465	1.744

74	1.315	2.485	1.745
75	1.319	2.480	1.745
76	1.309	2.450	1.742
77	1.324	2.492	1.744
78	1.314	2.460	1.742
79	1.305	2.461	1.743
80	1.345	2.534	1.748
81	1.339	2.540	1.747
82	1.344	2.546	1.744
83	1.301	2.437	1.741
84	1.323	2.490	1.744
85	1.320	2.495	1.743
86	1.309	2.447	1.740
87	1.322	2.491	1.744
88	1.352	2.550	1.748
89	1.336	2.506	1.744
90	1.306	2.442	1.741
91	1.334	2.510	1.745
92	1.322	2.485	1.745
93	1.323	2.481	1.745
94	1.330	2.497	1.748
95	1.327	2.499	1.745
96	1.322	2.476	1.744
97	1.323	2.471	1.747
98	1.342	2.525	1.749
99	1.335	2.503	1.746
100	1.329	2.506	1.744
**Average**	1.301	2.451	1.741
**Std Dev**	0.030	0.046	0.004

Legends for the Supplementary Movies

Supplementary Movie 1. A crystal of **APO@1** emitting light of different colors (orange, green, blue), depending on the excitation wavelength. The first image is from the crystal under white light.

Supplementary Movie 2. A pair of micromanipulators twinning and detwinning a crystal under polarized white light and UV light.

Supplementary Movie 3. Fluorescence recovery over time of each domain (parent, twinned, detwinned) monitored under excitation with 330–380 nm, 405 nm, and 488 nm light. The time is displayed in the upper right in hh:mm (hour:minute) format.

Supplementary Movie 4. Force/emission experiment measuring the color of light emitted in area 1 and area 2 of the crystal as it undergoes twin formation.
